# Brown-fat-mediated tumour suppression by cold-altered global metabolism

**DOI:** 10.1038/s41586-022-05030-3

**Published:** 2022-08-03

**Authors:** Takahiro Seki, Yunlong Yang, Xiaoting Sun, Sharon Lim, Sisi Xie, Ziheng Guo, Wenjing Xiong, Masashi Kuroda, Hiroshi Sakaue, Kayoko Hosaka, Xu Jing, Masahito Yoshihara, Lili Qu, Xin Li, Yuguo Chen, Yihai Cao

**Affiliations:** 1 Department of Microbiology, Tumor and Cell Biology, Karolinska Institutet, Solna, Sweden; 2 Department of Cellular and Genetic Medicine, School of Basic Medical Sciences, Fudan University, Shanghai, China; 3 Oujiang Laboratory (Zhejiang Lab for Regenerative Medicine, Vison and Brain Health), School of Pharmaceutical Science, Wenzhou Medical University, Wenzhou, China; 4 Longyan First Hospital Affiliated to Fujian Medical University, Longyan, China; 5 Department of Pancreatic Surgery, West China Hospital, Sichuan University, Chengdu, China; 6 Department Nuclear Medicine, Department of Emergency Medicine, Shandong Provincial Clinical Research Center for Emergency and Critical Care Medicine, Institute of Emergency and Critical Care Medicine of Shandong University, Qilu Hospital of Shandong University, Jinan, China; 7 Department of Nutrition and Metabolism, Tokushima University Graduate School, Tokushima, Japan; 8 Department of Head and Neck Surgery, Center of Otolaryngology-Head and Neck Surgery, Zhejiang Provincial People’s Hospital, People’s Hospital of Hangzhou Medical College, Hangzhou, China; 9 Department of Biosciences and Nutrition, Karolinska Institutet, Huddinge, Sweden

**Keywords:** Cancer metabolism, Translational research, Cancer therapy

## Abstract

Glucose uptake is essential for cancer glycolysis and is involved in non-shivering thermogenesis of adipose tissues^1–6^. Most cancers use glycolysis to harness energy for their infinite growth, invasion and metastasis^2,7,8^. Activation of thermogenic metabolism in brown adipose tissue (BAT) by cold and drugs instigates blood glucose uptake in adipocytes^4,5,9^. However, the functional effects of the global metabolic changes associated with BAT activation on tumour growth are unclear. Here we show that exposure of tumour-bearing mice to cold conditions markedly inhibits the growth of various types of solid tumours, including clinically untreatable cancers such as pancreatic cancers. Mechanistically, cold-induced BAT activation substantially decreases blood glucose and impedes the glycolysis-based metabolism in cancer cells. The removal of BAT and feeding on a high-glucose diet under cold exposure restore tumour growth, and genetic deletion of *Ucp1*—the key mediator for BAT-thermogenesis—ablates the cold-triggered anticancer effect. In a pilot human study, mild cold exposure activates a substantial amount of BAT in both healthy humans and a patient with cancer with mitigated glucose uptake in the tumour tissue. These findings provide a previously undescribed concept and paradigm for cancer therapy that uses a simple and effective approach. We anticipate that cold exposure and activation of BAT through any other approach, such as drugs and devices either alone or in combination with other anticancer therapeutics, will provide a general approach for the effective treatment of various cancers.

## Abstract (web summary)

Mild cold exposure activates a substantial amount of brown adipose tissue (BAT) in a patient with cancer, reducing tumour-associated glucose uptake, and activation of BAT in mice inhibits the growth of tumours by decreasing blood glucose and impeding glycolysis-based metabolism in cancer cells.

## Main

Metabolic alterations are one of the hallmarks of cancer and malignant cells often reprogram metabolism to promote their growth, proliferation, migration and survival^7,8,10,11^. A distinctive feature of altered metabolism commonly shared by most cancers is the high uptake of glucose that undergoes fermentation metabolism to generate lactate^1,12^. Under aerobic conditions, cancer cells use the glucose-fermentation–lactate pathway to harness energy in the form of ATP^2,13^. This cancer-associated aerobic glycolysis, that is, the Warburg effect, is an inefficient pathway to generate ATP^13^. Compared with mitochondrial respiration, the Warburg effect is the default pathway for energy production and only two ATP molecules are generated per glucose through aerobic glycolysis^1,13^. However, a 10–100-fold accelerated rate of glucose-based aerobic glycolysis occurs in tumour cells relative to that of glucose complete oxidation in the mitochondria of healthy cells^14^. Indeed, over 70% of human cancers show amplified expression of glycolytic genes^15–17^.

High rates of aerobic glycolysis results in cancer cells taking up more glucose from the tumour microenvironment (TME) and producing high amounts of lactic acids^13^. Glucose uptake is a rate-limiting step of aerobic glycolysis and is mediated by glucose transporters (GLUTs) in the cancer cell membrane^18,19^. Along with tumour growth, hypoxia becomes ostensible and hypoxia upregulates GLUT1, the key GLUT expressed in cancer cells, through hypoxia-inducible factor-1α (HIF-1α)-triggered transcriptional regulation^20^. Aerobic glycolysis provides abundant metabolites as intermediates for macromolecule biosynthesis, including the nucleotide substrates ribose-5-phosphate and glycine as well as the lipid substrate glycerol^21^. Accumulation of intracellular lactic acids facilitates their secretion through the proton-linked monocarboxylate transporter, causing a high grade of acidosis in the TME^22,23^.

BAT is a specialized tissue for energy expenditure by generating heat^4^. Cold-acclimatization-, diet- and drug-triggered sympathetic activation induces BAT activation and the conversion of white adipose tissue (WAT) to a brown-like phenotype^5,6,24–27^. Accordingly, activated BAT and browning WAT generate heat through non-shivering thermogenesis (NST)^28,29^, which is mediated by uncoupling protein 1 (UCP1) expressed in the inner membrane of the mitochondria^30^. Accumulating evidence shows that glucose contributes to the  BAT thermogenesis and genetic knockdown of *Glut1* and *Glut4* or hexokinase (the initial enzyme in glycolysis) markedly impairs metabolism^9^. BAT-mediated NST is an effective energy-expenditure mechanism for reducing body weight and improving metabolic dysfunctions in obese and diabetic animals^4,26,27^. As a substantial amount of BAT tissue mass exists in adult humans, it is speculated that activation of BAT thermogenesis would provide an attractive approach for treating obesity and type 2 diabetes mellitus^4,26,27^.

Here we show that BAT activation induced by cold acclimatization markedly inhibited the growth of various solid tumours, dependent on UCP1 thermogenesis. BAT removal and genetic deletion of *Ucp1* restored the tumour growth rate under cold exposure. We provide mechanistic insights into NST-triggered tumour suppression. Finally, we provide preliminary findings of BAT activation in both adult healthy humans and a patient with cancer by mild cold exposure. Cold exposure also markedly reduced glucose uptake in a human tumour. Our discoveries provide a concept for cancer treatment and will offer substantial clinical benefits for patients with cancer.

## Cold-induced tumour inhibition and survival

Malignancy is considered to be a metabolic disorder and most solid tumours acquire their energy for growth and progression through accelerated glycolytic metabolism, that is, the Warburg effect^13^. However, alterations of the TME and the host macroenvironment often engender metabolic reprogramming of cancer cells. In particular, activation of lipid metabolism may substantially reprogram cancer metabolic pathways and, as a consequence, alter tumour growth rates, metastatic potentials and drug responses^31–34^. To study the effect of thermogenesis-related metabolism in adipose tissues on tumour growth and the survival of hosts with cancer, we examined the effect of cold acclimatization, which is known to activate BAT^35^, on tumour growth and progression.

**Fig. 1:**
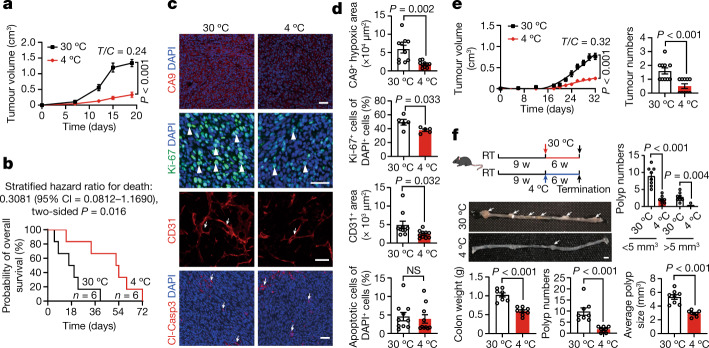
Cold exposure suppresses xenograft and genetic spontaneous tumour growth, prolongs the overall survival of tumour-bearing mice and alters the TME. **a**, Tumour growth of mouse CRC under 30 °C and 4 °C conditions. *n* = 8 mice per group. *T*/*C* = ratio of tumour growth in the treated group versus control group. **b**, Overall survival of CRC-tumour-bearing mice. *n* = 6 mice per group. **c**, Immunofluorescence staining of CRC tumours for CA9^+^ hypoxic area, Ki-67^+^ proliferating cells, CD31^+^ microvessels and cleaved caspase 3 (Cl-Casp3)^+^ apoptotic cells. The arrows and arrowheads indicate the respective positive signals. Tumour tissues were counterstained with 4′,6-diamidino-2-phenylindole (DAPI). Scale bars, 50 μm. **d**, Quantitative analysis of the positive signals shown in **c**. *n* = 5 or 10 random fields per group. **e**, Tumour growth rates and tumour incidences of spontaneous breast cancer in the MMTV-PyMT model under the 30 °C and 4 °C conditions. *n* = 10 mice per group. **f**, Experimental schematic, intestinal adenoma morphology, colon weights, polyp numbers, average polyp sizes and polyp size distribution in the *Apc*^*min/+*^ model under the 30 °C and 4 °C conditions. *n* = 8 mice per group. The arrows indicate polyps. Scale bar, 5 mm. RT, room temperature; w, weeks. For **a** and **d–f**, data are mean ± s.e.m. For **a** and **d**–**f**, statistical analysis was performed using two-sided unpaired *t*-tests. NS, not significant. Source data

**Extended Data Fig. 1:**
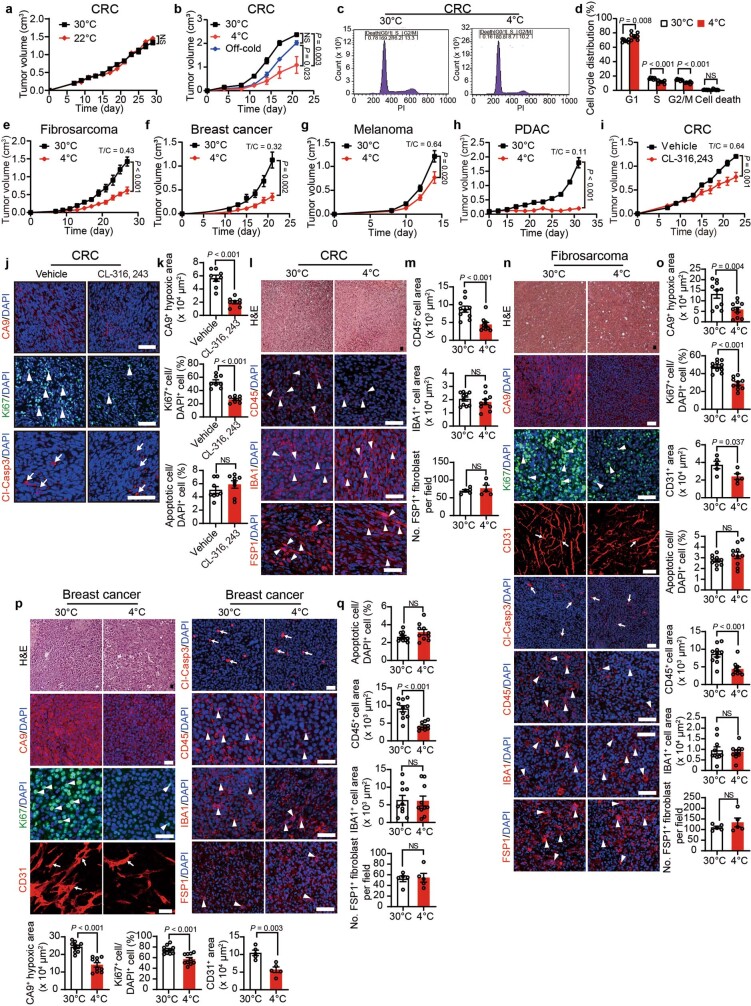
Reversible tumour suppression by off-cold and changes of tumour microenvironment by cold exposure. **a**, **b**, Tumour growth of CRC under 30 °C and 22 °C (**a**), under 30 °C, 4 °C, and 4 °C for 11 days followed by switching to 30 °C for 10 days (**b**) (*n* = 8 mice per group in **a** and 5 mice per group in **b**). **c**, **d**, Representative FACS and quantification of CRC tumour cells under 30 °C and 4 °C for cell cycle analysis (*n* = 7 tumours per group). **e**–**h**, Tumour growth of murine fibrosarcoma (**e**), murine breast cancer (**f**), murine melanoma (**g**), and murine pancreatic ductal adenocarcinoma (PDAC) (**h**) under 30 °C and 4 °C (*n* = 8 mice per group in **e**, **g**, **h** and 6 mice per group in **f**). **i**, Tumour growth of vehicle- and CL-316,243-treated CRC under 30 °C and 4 °C (*n* = 6 mice per group). **j**, **k**, Immunofluorescence staining and quantifications of CA9^+^ hypoxic area, Ki-67^+^ proliferating cells, Cl-Casp3^+^ apoptotic cells. Arrows and arrowheads point to positive signals. Tumour tissues were counterstained with DAPI (blue). **l**–**q**, Immunofluorescence staining and quantifications of morphology (H&E), CA9^+^ hypoxic area, Ki-67^+^ proliferating cells, CD31^+^ microvessels, Cl-Casp3^+^ apoptotic cells, CD45^+^ inflammatory myeloid cells, IBA1^+^ pan-macrophages, and FSP1^+^ fibroblasts in CRC, fibrosarcoma, and breast cancer under 30 °C and 4 °C (*n* = 5 or 10 random fields per group). Arrows and arrowheads point to positive signals. All scale bars, 50 μm. Data presented as mean ± s.e.m. Statistical analysis was performed using one-way ANOVA followed by Tukey's multiple comparisons test (**b**), or two-sided unpaired *t*-test (**a**, **d**, **e**–**i**, **k**, **m**, **o**, **q**). NS, not significant. Source data

Subcutaneous implantation of colorectal cancer (CRC) into immunocompetent C57BL/6 mice resulted in marked inhibition of tumour growth under 4 °C relative to those under 30 °C (Fig. 1a). The cold-exposure-induced tumour suppression was robust and approximately 80% inhibition was recorded on day 20 after tumour implantation (Fig. 1a). The anticancer effect was not observed at 22 °C (Extended Data Fig. 1a), which is a commonly used temperature for housing experimental mice. Inhibition of tumour growth by cold exposure was reversible after cessation of low temperature (Extended Data Fig. 1b). Reconciling with this notion, the subpopulation of tumour cells at the G0/G1 phase was significantly increased, whereas cell populations in the S and G2/M phases decreased under 4 °C (Extended Data Fig. 1c,d). To generalize these findings, we studied cold-induced tumour suppression in other cancer types, including fibrosarcoma, breast cancer, melanoma and pancreatic ductal adenocarcinoma. Similarly, cold exposure of tumour-bearing mice also markedly inhibited the growth rates of these tumour types (Extended Data Fig. 1e–h). These findings provide compelling evidence that cold exposure significantly inhibited the growth rates of various tumour types in mice. Consistent with delayed tumour growth rates, the survival of CRC tumour-bearing mice was markedly prolonged under cold acclimatization (Fig. 1b). Compared with the 30 °C thermoneutral condition, the overall survival of tumour-bearing mice exposed to 4 °C was almost doubled. In addition to cold exposure, we used a β3-adrenoceptor agonist CL-316,243 to treat CRC tumour-bearing mice to further validate our findings. Expectedly, CL-316,243 also markedly inhibited tumour growth and tumour cell proliferation in vivo (Extended Data Fig. 1i–k).

**Extended Data Fig. 2:**
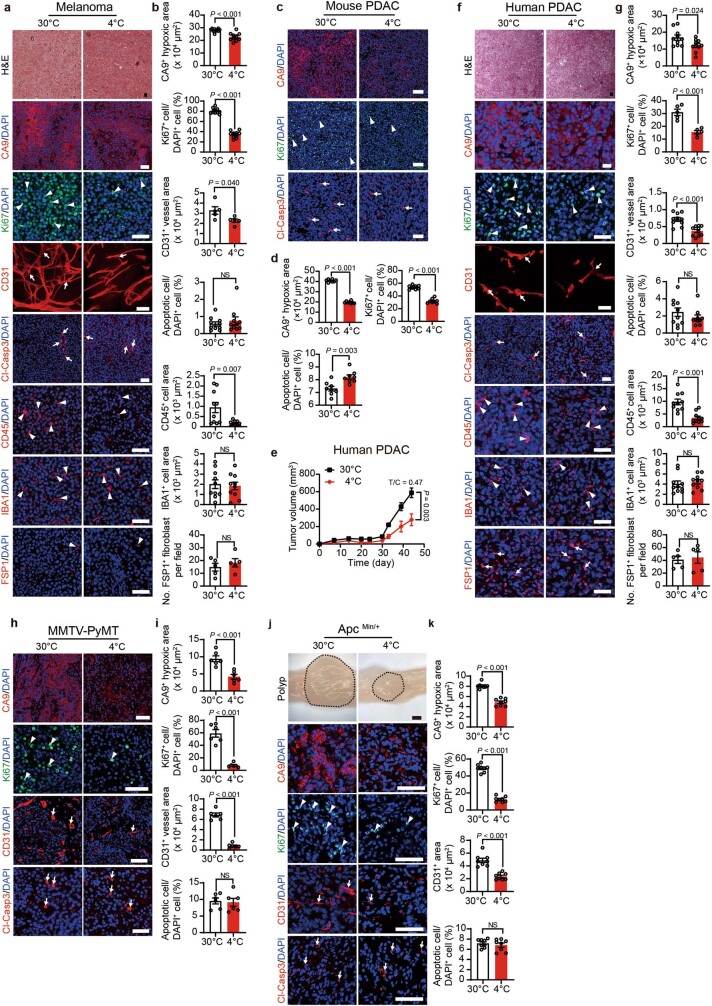
Changes of the tumour microenvironment by cold in various tumour models. **a**–**g**, Immunofluorescence staining and quantifications of morphology (H&E), CA9^+^ hypoxic area, Ki-67^+^ proliferating cells, CD31^+^ microvessels, Cl-Casp3^+^ apoptotic cells, CD45^+^ inflammatory myeloid cells, IBA1^+^ pan-macrophages, and FSP1^+^ fibroblasts in melanoma (**a**, **b**), mouse PDAC (**c**, **d**), and human PDAC (**f**, **g**) under 30 °C and 4 °C (*n* = 5, 8, or 10 random fields per group). Arrows and arrowheads point to positive signals. Tumour growth of human PDAC (**e**) under 30 °C and 4 °C (*n* = 8 mice per group). **h**–**k**, Immunofluorescence staining and quantifications of CA9^+^ hypoxic area, Ki-67^+^ proliferating cells, CD31^+^ microvessels, and Cl-Casp3^+^ apoptotic cells in MMTV-PyMT tumours **(h**, **i**), and Apc^Min/+^ polyps (**j**, **k**) under 30 °C and 4 °C (*n* = 6 or 8 random fields per group). Arrows and arrowheads point to positive signals. Scale bars in the upper panel of **j**, 1 mm. All other scale bars, 50 μm. Data presented as mean ± s.e.m. Statistical analysis was performed using two-sided unpaired *t*-test (**b**, **d**, **e**, **g**, **i**, **k**). NS, not significant. Source data

To investigate the alteration of the TME under the 30 °C thermoneutrality and 4 °C cold exposure conditions, we performed immunohistochemical analysis using multiple markers to detect various cellular components^3,36–41^. Interestingly, cold exposure improved hypoxia in the CRC tumours (Fig. 1c,d). The density of CD31^+^ tumour microvessels was also significantly reduced under cold acclimatization despite the approximately equal tumour size in the cold and thermoneutral groups (Fig. 1c,d). Importantly, the proliferative rate of tumour cells as measured by Ki-67 was substantially inhibited by cold exposure. Moreover, the CD45^+^ myeloid cell population was decreased in cold-exposed CRC tumours relative to 30 °C-exposed tumours (Extended Data Fig. 1l,m). By contrast, the pan population of IBA1^+^ tumour-associated macrophages and fibroblast specific protein 1 (FSP1)-positive cancer-associated fibroblasts remained unchanged (Extended Data Fig. 1l,m). No alterations in cellular apoptosis were observed (Fig. 1c,d). Immunohistochemistry analysis of the cold-exposed fibrosarcoma, breast cancer, melanoma and pancreatic cancer produced nearly identical results (Extended Data Figs. 1n–q and 2a–g).

To further corroborate these findings, two genetic spontaneous tumour models were used for cold exposure studies. In the MMTV-PyMT breast cancer model, exposure of mice (aged 10 weeks) to 4 °C markedly inhibited tumour growth incidence (Fig. 1e). Similar to xenograft tumours, the spontaneous MMTV-PyMT tumour cells proliferated at low rates under cold exposure relative to those under thermoneutrality (Extended Data Fig. 2h,i). CD31^+^ vessel structures and tumour hypoxia were decreased (Extended Data Fig. 2h,i). In the spontaneous *Apc*^*Min/+*^ intestinal adenoma model, cold exposure suppressed tumour formation and tumour growth (Fig. 1f). Similar to MMTV breast cancers, tumour cell proliferation, tumour hypoxia and angiogenesis were inhibited by cold (Extended Data Fig. 2j,k). These genetic mouse tumour models further validate the cold-triggered tumour suppression. Together, these results show that cold exposure significantly inhibits tumour growth and alters the stromal cellular components in TME.

## Tumour inhibition in the liver by cold exposure

**Extended Data Fig. 3:**
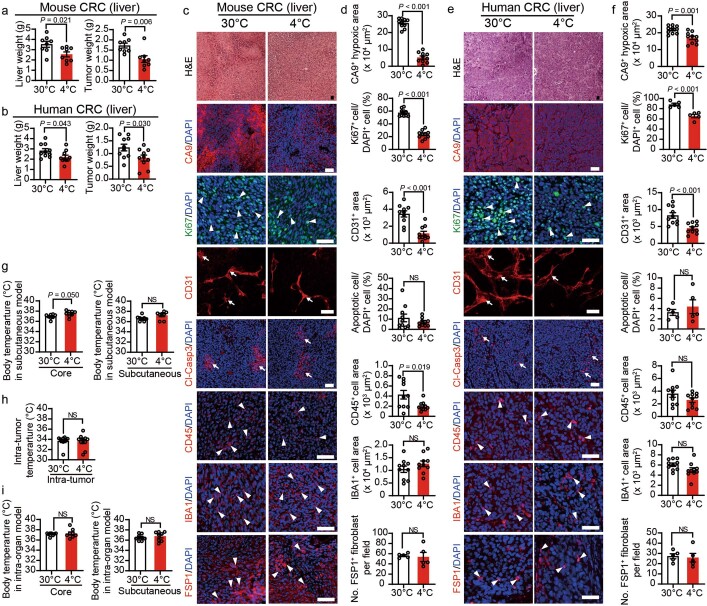
Cold exposure inhibits tumour growth in a liver tumour model. **a**, Total liver (left) and tumour (right) weights of liver murine CRC tumour-bearing mice under 30 °C and 4 °C (*n* = 8 mice per group). **b**, Total liver (left) and tumour (right) weights of liver human CRC tumour-bearing immunodeficient mice under 30 °C and 4 °C (*n* = 10 mice per group). **c**-**f**, Immunofluorescence staining and quantifications of morphology (H&E), CA9^+^ hypoxic area, Ki-67^+^ proliferating cells, CD31^+^ microvessels, Cl-Casp3^+^ apoptotic cells, CD45^+^ inflammatory myeloid cells, IBA1^+^ pan-macrophages, and FSP1^+^ fibroblasts in liver murine CRC (**c**, **d**) and liver human CRC (**e**, **f**) under 30 °C and 4 °C (*n* = 5 or 10 random fields per group). Arrows and arrowheads point to positive signals. **g**, Core and subcutaneous body temperatures of murine CRC subcutaneous tumour-bearing mice under 30 °C and 4 °C (*n* = 8 mice per group). **h**, Intra-tumoral temperature of subcutaneous murine CRC tumours under 30 °C and 4 °C (*n* = 10 tumours per group). **i**, Core (left) and subcutaneous (right) body temperature of murine CRC tumour-bearing mice in the intra-liver implantation model under 30 °C or 4 °C (*n* = 8 mice per group). All scale bars, 50 μm. Data presented as mean ± s.e.m. Statistical analysis was performed using two-sided unpaired *t*-test (**a**, **b**, **d**, **f**, **g**–**i**). NS, not significant. Source data

In the subcutaneous tumour model, there was a possibility that cold exposure inhibited tumour growth through reducing the local temperature in the subcutaneous location. Although the spontaneous *Apc*^*Min/+*^ intestinal adenoma model largely excluded this probability, we implanted tumours into internal organs to further exclude this possibility. We chose the liver as a site for implantation because CRCs often metastasize to the liver tissue. Similar to the subcutaneous tumour model, implantation of mouse CRC into the liver resulted in marked inhibition of tumour growth under cold acclimatization (Extended Data Fig. 3a). Similarly, implantation of a human CRC in the immunodeficient mice also reproduced the cold-triggered tumour suppression in the liver tissue (Extended Data Fig. 3b). These findings excluded the possibility of the direct effect of low temperature by skin contact in cold-induced tumour suppression.

Immunohistochemistry analysis of mouse and human CRC tumour tissues validated the findings described in the subcutaneous xenograft and genetic tumour models, including alleviation of tumour hypoxia, reduction of CD31^+^ microvessels, reduced tumour cell proliferation and decreased CD45^+^ populations (Extended Data Fig. 3c–f). These data validate the fact that cold-induced tumour suppression occurs in a tissue- or organ-independent manner.

To further exclude the effect of low-temperature contact on tumour suppression, we measured the body temperature of tumour-bearing mice in different locations. Under 4 °C exposure, the core body temperature (CBT) as measured in the anus was increased compared with that under the 30 °C condition (Extended Data Fig. 3g). Insertion of the probe in the subcutaneous region did not detect any difference in body temperature (Extended Data Fig. 3g). Furthermore, the temperature in the tumour tissue remained unchanged between the 4 °C- and 30 °C-exposed tumour-bearing mice (Extended Data Fig. 3h). Similarly, the CBT and subcutaneous temperature in the intraorgan model were nearly identical under thermoneutral and cold conditions (Extended Data Fig. 3i). These data further exclude the possibility that direct contact of low temperature influences the delayed tumour growth rates.

## BAT and subcutaneous WAT browning by cold

**Fig. 2:**
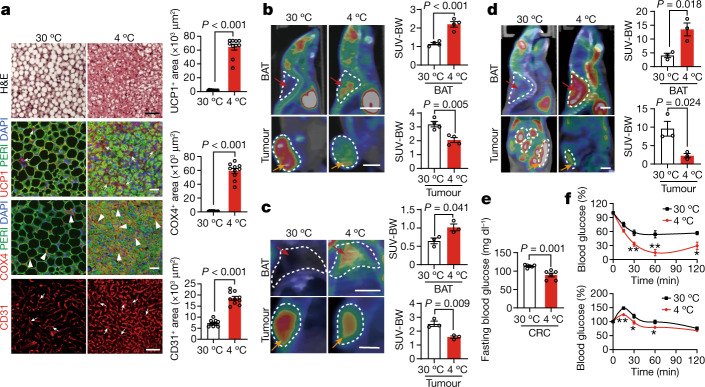
Adipose activation and glucose uptake in adipose tissues and tumours. **a**, Histological and immunofluorescence staining of BAT for UCP1, perilipin, COX4 and CD31, followed by counterstaining with DAPI (blue) in CRC-tumour-bearing mice under the 30 °C and 4 °C conditions. The arrows and arrowheads indicate positive signals. Positive signals were randomly quantified. *n* = 10 random fields per group. Scale bars, 50 μm. **b**–**d**, Representative PET–CT images of BATs and tumours and quantification of standardized uptake values (SUV) normalized to body weight (SUV-BW) of mice bearing mouse CRC tumours of similar tumour size (**b**), MMTV-PyMT mice with similar tumour size (**c**) and *Apc*^*Min/+*^ mice with similar age (**d**) under the 30 °C and 4 °C conditions. *n* = 4 (**b**) and *n* = 3 (**c** and **d**) mice per group. BATs and tumours are indicated by the red and orange arrows, respectively. For **b**–**d**, scale bars, 5 mm. **e**, Fasting blood glucose concentrations of CRC-tumour-bearing mice under the 30 °C and 4 °C conditions. *n* = 6 mice per group. **f**, Insulin-tolerance test (top) and glucose-tolerance test (bottom) of CRC-tumour-bearing mice under the 30 °C and 4 °C conditions. *n* = 6 mice per group. For **a**–**f**, data are mean ± s.e.m. Statistical analysis was performed using two-sided unpaired *t*-tests (**a**–**f**). *P < 0.05, **P < 0.01. Exact *P* values are provided in the Source Data.Source Data Source data

**Extended Data Fig. 4:**
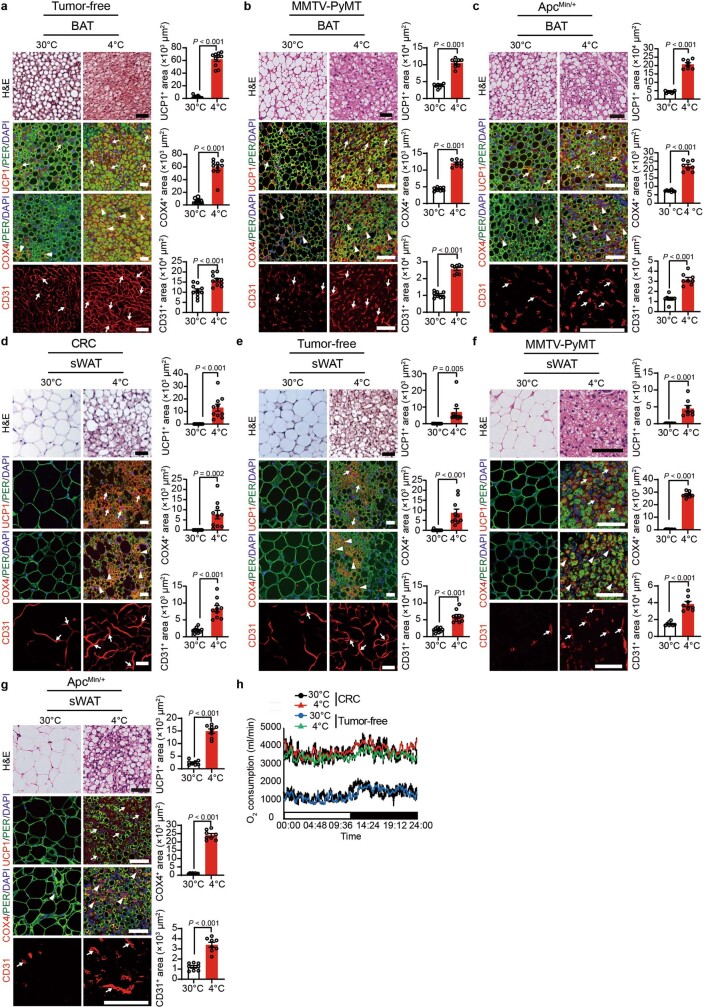
Browning of adipose tissues by cold in xenograft and genetically spontaneous tumour models. **a**–**c**, Histological and immunofluorescence staining of BAT in tumour-free mice (**a**), MMTV-PyMT tumour-bearing mice (**b**), and Apc^Min/+^ mice (**c**) under 30 °C and 4 °C with H&E, UCP1, perilipin, COX4, and CD31, followed by counterstaining with DAPI (blue). Positive signals were randomly quantified (*n* = 8 or 10 random fields per group). Arrows and arrowheads point to positive signals. **d**–**g**, Histological and immunofluorescence staining of sWAT in CRC tumour-bearing mice (**d**), tumour-free mice (**e**), MMTV-PyMT tumour-bearing mice (**f**), and Apc^Min/+^ mice (**g**) under 30 °C and 4 °C with H&E, UCP1, perilipin, COX4, and CD31, followed by counterstaining with DAPI (blue). Positive signals were randomly quantified (*n* = 8 or 10 random fields per group). Arrows and arrowheads point to positive signals. **h**, Whole-body metabolism of CRC tumour-bearing and tumour-free mice under 30 °C and 4 °C (n = 3 mice per group). All scale bars, 50 μm. Data presented as mean ± s.e.m. Statistical analysis was performed using two-sided unpaired *t*-test (**a**–**g**). Source data

To study whether cold exposure could activate BAT in tumour-bearing mice, tumour-free and tumour-bearing mice were exposed to 4 °C and 30 °C. Similar to tumour-free mice, the BAT in the xenograft and genetically spontaneous tumour-bearing mice exhibited highly dense structures, which appeared as smaller multilocular structures (Fig. 2a and Extended Data Fig. 4a–c), a typical morphological phenotype for BAT activation. Consistent with phenotypical alterations, the cytochrome *c* oxidase subunit 4 (COX4)^+^ mitochondrion contents and CD31^+^ microvessel density were markedly increased in the BAT under cold acclimatization (Fig. 2a and Extended Data Fig. 4a–c). Expression levels of UCP1, a key thermogenic protein, were accordingly increased in tumour-bearing mice as observed in tumour-free mice (Fig. 2a and Extended Data Fig. 4a–c). These findings indicate that the implantation of tumours into mice did not significantly affect cold-augmented BAT activation.

It is known that cold exposure also activates WAT, especially subcutaneous WAT (sWAT), and exhibits a browning phenotype^29^. Histology and immunohistochemical examination of sWAT demonstrated a typical browning phenotype, including smaller adipocytes, enrichment of intracellular multivesicular structures, high contents of mitochondria, increased numbers of microvessels and elevated UCP1 expression in both xenograft and spontaneous tumour models (Extended Data Fig. 4d–g). These data indicate that tumours do not interfere with sWAT browning.

We next used positron emission tomography–computed tomography (PET–CT) imaging analysis by measuring the uptake of ^18^F-fluorodeoxyglucose (^18^F-FDG) in various organs of tumour-bearing mice. Under thermoneutral conditions, ^18^F-FDG distribution mainly accumulated in the tumour tissues and modest signals were found in the BAT in the xenograft and spontaneous tumour models (Fig. 2b–d). However, cold exposure markedly instigated ^18^F-FDG distribution in the interscapular BAT (iBAT) and ^18^F-FDG signals were barely detectable in tumours despite the approximately equal tumour sizes in the cold and thermoneutral groups (Fig. 2b–d). Note that xenograft tumour-bearing mice under cold acclimatization were prolonged for experimentation duration to allow the tumours to reach the same size as in the thermoneutral group. Quantitative analysis further supports the conclusion of cold-induced redistribution of ^18^F-FDG between iBAT and tumours (Fig. 2b–d). These findings indicate that cold exposure reduces glucose uptake in tumours by instigating BAT glucose uptake.

## BAT-dependent suppression of tumour growth

**Extended Data Fig. 5:**
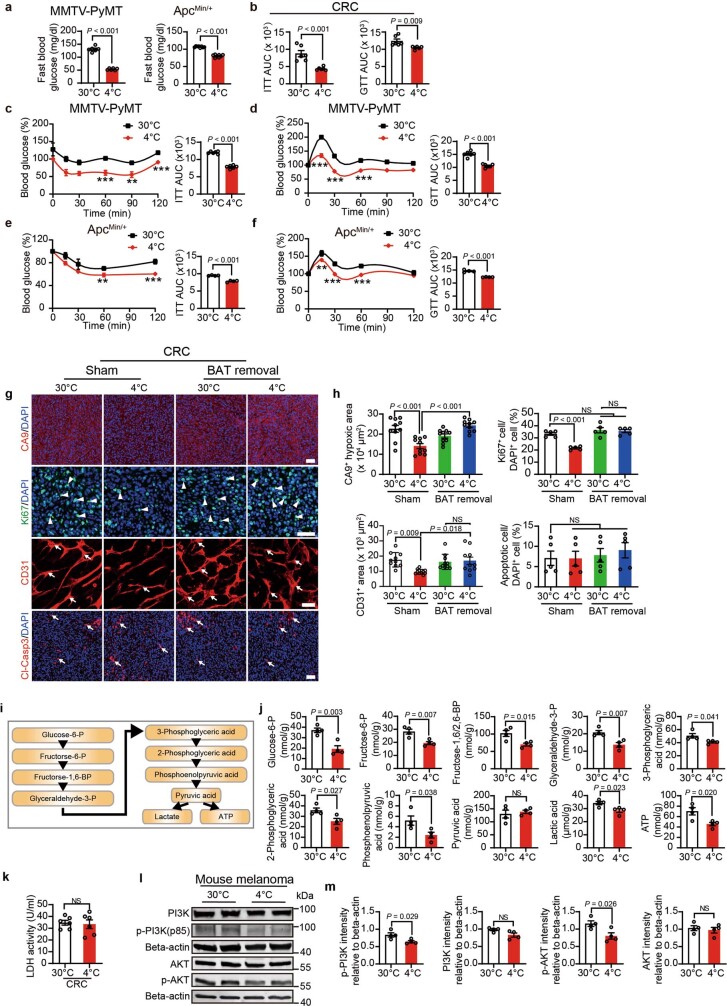
Removal of BAT ablates tumour microenvironmental changes and the mitigation of PI3K activation in cold-exposed tumours. **a**, Fasting blood glucose concentrations of MMTV-PyMT tumour-bearing mice and Apc^Min/+^ mice under 30 °C and 4 °C (*n* = 6 mice per group). **b**–**f**, Insulin tolerance test and glucose tolerance test of CRC tumour-bearing mice (**b**), MMTV-PyMT tumour-bearing mice (**c**, **d**) and Apc^Min/+^ mice (**e**, **f**) under 30 °C and 4 °C (*n* = 6 mice per group in **b**-**d** and 4 mice per group in **e**, **f**). **g**, **h**, Immunofluorescence staining and quantifications of CA9^+^ hypoxic area, Ki-67^+^ proliferating cells, CD31^+^ microvessels, and Cl-Casp3^+^ apoptotic cells in sham-operated or BAT-removed CRC tumour-bearing mice under 30 °C and 4 °C (*n* = 5 or 10 random fields per group). Arrows and arrowheads point to positive signals. **i**, Schematic representation of the glycolysis pathway. **j**, Amounts of the glycolytic metabolites of CRC tumours under 30 °C and 4 °C (*n* = 4 biological samples per group). **k**, Quantification of LDH activity in CRC tumours under 30 °C and 4 °C (*n* = 6 tumours per group). **l**, **m**, Immunoblot analysis (**l**) and quantification (**m**) of non-phosphorylated and phosphorylated PI3K (p-PI3K) and AKT and phosphorylated AKT (p-AKT) in melanoma. Beta-actin was used for standardizing total protein loading levels (*n* = 4 biological samples per group). All scale bars, 50 μm. Data presented as mean ± s.e.m. Statistical analysis was performed using one-way ANOVA followed by Tukey’s multiple comparison tests (**h**) or two-sided unpaired *t*-test (**a**–**f**, **j**, **k**, **m**). NS, not significant; **P < 0.01; ***P < 0.001 (exact *P* values are presented in the source data). Source data

It is known that cold acclimatization accelerates thermogenic metabolism in mice^4,5^. To study the metabolic changes under cold exposure, thermogenic metabolism was measured in tumour-bearing mice. Despite the similar tumour size in the 4 °C and 30 °C groups, cold exposure increased the metabolism in CRC-tumour-bearing mice relative to the thermoneutral group (Extended Data Fig. 4h). Notably, the levels of fast blood glucose were significantly decreased in the cold-exposure tumour-bearing mice in the xenograft and spontaneous tumour models (Fig. 2e and Extended Data Fig. 5a). Insulin-tolerance and glucose-tolerance tests showed a marked improvement of insulin sensitivity and quick glucose clearance under cold exposure in the xenograft and spontaneous tumour models (Fig. 2f and Extended Data Fig. 5b–f). These experiments demonstrate that cold exposure significantly decreases blood glucose levels and improves insulin sensitivity in tumour-bearing mice.

**Fig. 3:**
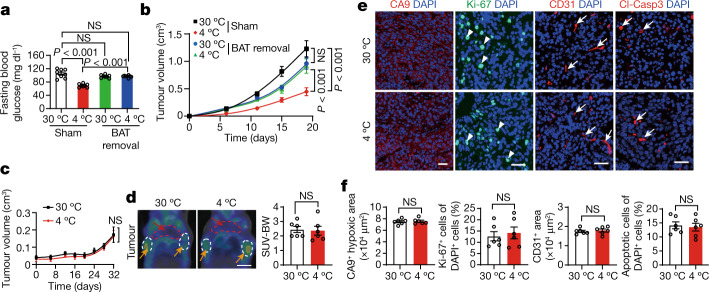
Removal of BAT ablates cold-triggered tumour suppression. **a**, Blood glucose levels of CRC-tumour-bearing mice after sham and BAT removal under 30 °C or 4 °C. *n* = 8 mice per group. **b**, CRC tumour growth rates in sham and BAT-removed mice under 30 °C or 4 °C conditions. *n* = 10 mice per group. **c**, Tumour growth curves of BAT-removed MMTV-PyMT mice under 30 °C and 4 °C conditions. *n* = 6 mice per group. **d**, Representative PET–CT images and quantification of SUV-BW of BAT-removed MMTV-PyMT mice under 30 °C or 4 °C conditions. *n* = 6 mice per group. Scale bar, 5 mm. **e**, Immunofluorescence staining of tumours grown in BAT-removed MMTV-PyMT mice with CA9^+^ hypoxic area, Ki-67^+^ proliferating cells, CD31^+^ microvessels and Cl-Casp3^+^ apoptotic cells. The arrows and arrowheads indicate the respective positive signals. Tumour tissues were counterstained with DAPI (blue). Scale bars, 50 μm. **f**, Quantification of positive signals of the markers presented in **e**. *n* = 6 random fields per group. For **a**–**d** and **f**, data are mean ± s.e.m. Statistical analysis was performed using one-way analysis of variance (ANOVA) followed by Tukey multiple-comparison test (**a** and **b**) and two-sided unpaired *t*-tests (**c**, **d** and **f**). Source data

To determine the role of BAT activation in tumour suppression, we next performed a surgical operation in tumour-bearing mice by removing BAT. Interestingly, removal of BAT significantly increased blood glucose levels under 4 °C (Fig. 3a), demonstrating that the activation of BAT was primarily responsible for blood glucose consumption. Importantly, the removal of BAT nearly abolished tumour suppression by cold acclimatization (Fig. 3b). By contrast, BAT removal had no effect on tumour growth under thermoneutral conditions (Fig. 3b). These results show that activation of BAT is primarily responsible for the cold-triggered tumour suppression. BAT removal also significantly improved tumour hypoxia, angiogenesis and tumour cell proliferation (Extended Data Fig. 5g,h). Thus, removal of BAT in cold-exposed tumour-bearing mice also markedly improved the TME, favouring tumour growth.

In addition to the xenograft CRC tumour model, BAT was also removed in spontaneous genetic MMTV-PyMT breast cancer tumour-bearing mice. Removal of BAT completely abolished cold-induced tumour suppression (Fig. 3c). Under equal tumour sizes, the cold-mitigated ^18^F-FDG uptake in breast cancers was completely neutralized by removing BAT, which was indistinguishable from the 30 °C-exposed group (Fig. 3d). Removal of BAT also ablated decreases in tumour hypoxia, angiogenesis and tumour cell proliferation by cold exposure (Fig. 3e,f). These data demonstrate a BAT-dependent mechanism of tumour suppression.

## Metabolic reprogramming in tumours

**Fig. 4:**
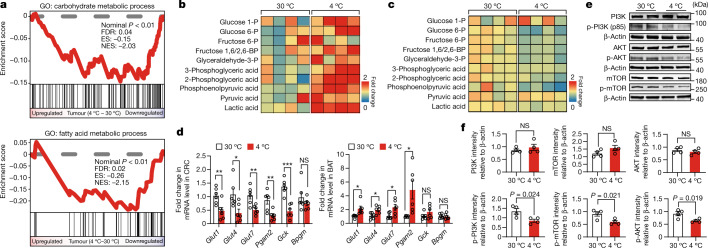
Metabolomic analysis of glycolysis and detection of PI3K signalling. **a**, GSEA comparing the expression of carbohydrate metabolic process (top) and fatty acid metabolic process (bottom) of CRC samples exposed to 30 °C and 4 °C. *n* = 3 and 2 biological samples per group, respectively. **b**,**c**, Metabolomic heat-map analysis of glycolysis-related metabolites of BAT (**b**) and tumours (**c**) of CRC-tumour-bearing mice under 30 °C and 4 °C conditions. Each column represents a biological sample. *n* = 4 biological samples per group. **d**, Quantitative PCR (qPCR) analysis of Glut genes and glycolysis-related genes in CRC tumour tissues. *n* = 6 samples per group. **e**,**f**, Immunoblot analysis (**e**) and quantification (**f**) of non-phosphorylated and phosphorylated PI3K, AKT and mTOR in CRC tumours exposed to 30 °C and 4 °C. *n* = 4 biological samples per group. β-Actin was used for standardizing total protein loading levels. For **d** and **f**, data are mean ± s.e.m. Statistical analysis was performed using Wald tests (**a**) or two-sided unpaired *t*-tests (**d** and **f**); *P < 0.05, **P < 0.01, ***P < 0.001. Exact *P* values are provided in the Source Data.Source Data Source data

Ablation of cold-induced tumour suppression by deleting BAT suggested to us that BAT activation by cold conditions could potentially alter tumour metabolism. To investigate this possibility, we used two unbiased genomic and proteomic approaches. Gene set enrichment analysis (GSEA) by RNA-sequencing (RNA-seq) showed that both glycolytic and lipid metabolisms in cold-exposed CRC tumours were attenuated (Fig. 4a). Detailed metabolomics analysis showed marked increases in glycolysis in the BAT of cold-exposed CRC tumour-bearing mice (Fig. 4b and Extended Data Fig. 5i). Several crucial components of the glycolytic pathway, including glucose 1-phosphate, glucose 6-phosphate, fructose 1,6-bisphosphate, glyceraldehyde-3-phosphate, 3-phosphoglyceric acid, 2-phosphoglyceric acid, phosphoenolpyruvic acid, pyruvic acid and lactate, were markedly increased (Fig. 4b). By contrast, these key glycolytic components were significantly decreased in tumours under cold exposure (Fig. 4c and Extended Data Fig. 5j). To exclude the possibility that mitigating lactate dehydrogenase (LDH) activity by cold exposure is responsible for the reduced lactate production, we measured the LDH activity of CRC tumours under 4 °C and 30 °C conditions. LDH activity was not altered under cold exposure (Extended Data Fig. 5k).

In particular, the levels of GLUTs, including *Glut1*, *Glut4* and *Glut7*, were markedly decreased in cold-exposed tumours (Fig. 4d). By contrast, *Glut4* and glycolysis-related genes were markedly elevated in the BAT under cold exposure (Fig. 4d). Consistent with suppression of tumour glycolysis, activation of phosphatidylinositol-3-kinase (PI3K), AKT and mammalian target of rapamycin (mTOR) in CRC tumours was also markedly inhibited by cold exposure (Fig. 4e,f). Similar inhibition of PI3K activation was also found in cold-exposed melanoma (Extended Data Fig. 5l,m). These data show that cold exposure induces metabolic reprogramming in tumours by suppressing the glycolytic pathway, which is essential for energy production and supporting tumour growth.

## High-glucose feeding restores tumour growth

**Extended Data Fig. 6:**
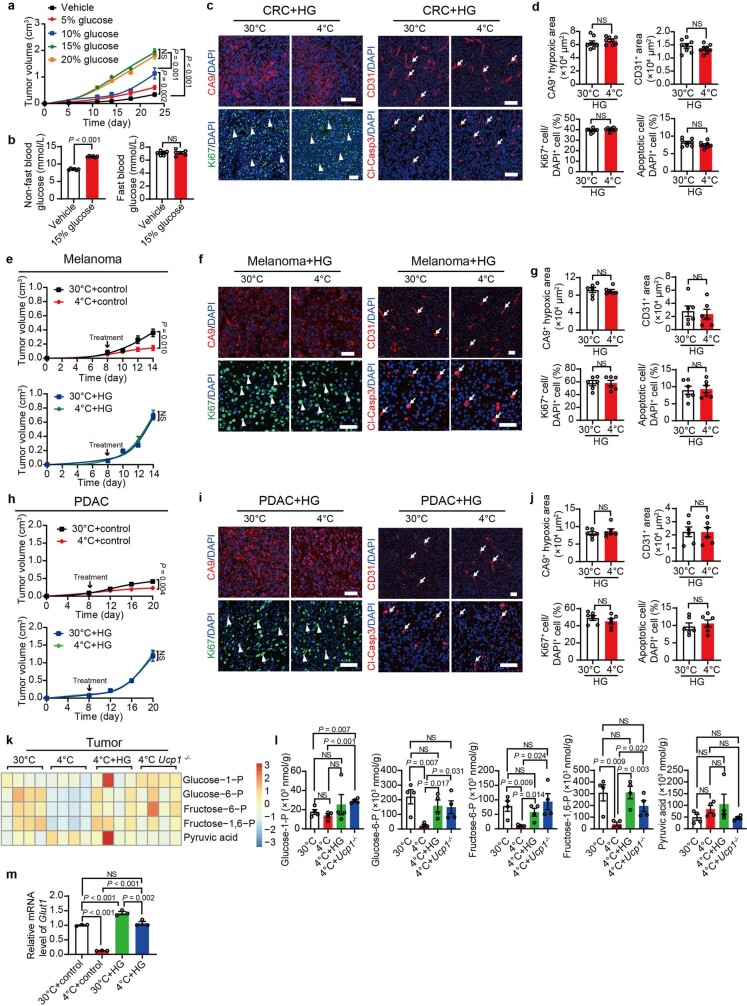
The impacts of high glucose feeding in glycolytic metabolism and tumour microenvironment. **a**, Tumour growth in subcutaneous murine CRC-bearing mice fed with various doses of glucose (*n* = 6 mice per group). **b**, Blood glucose levels of fasting (left) and non-fasting (right) mice receiving vehicle and 15% glucose solution (*n* = 6 mice per group). **c**-**j**, Immunofluorescence staining and quantifications of CA9^+^ hypoxic area, Ki-67^+^ proliferating cells, CD31^+^ microvessels, and Cl-Casp3^+^ apoptotic cells in HG-treated CRC (**c**, **d**), HG-treated melanoma (**f**, **g**), and HG-treated PDAC (**i**, **j**) under 30 °C and 4 °C (*n* = 6 or 8 random fields per group). Arrows and arrowheads point to positive signals. Tumour growth rates of vehicle-fed and 15% glucose-treated melanoma (**e**) or PDAC (**h**) under 30 °C or 4 °C (*n* = 5 mice per group). **k**, Metabolomic heatmap analysis of glycolysis-related metabolites of vehicle- and HG-treated CRC tumours grown in WT or *Ucp1*^−/−^ under 30 °C or 4 °C. **l**, Measurements of the amounts of glycolytic metabolites (*n* = 4 biological samples per group). **m**, qPCR analysis of *Glut1* of vehicle- and HG-treated CRC under 30 °C or 4 °C (*n* = 3 samples per group). All scale bars, 50 μm. Data presented as mean ± s.e.m. Statistical analysis was performed using one-way ANOVA followed by Tukey multiple tests (**a**, **m**) or two-sided unpaired *t*-test (**b**, **d**, **e**, **g**, **h**, **j**, **l**). HG, high glucose (15% glucose); NS, not significant. Source data

To test whether the hypothesis of blood glucose competition between tumours and activated BAT is the key for cold-induced tumour growth suppression, we implemented high-glucose feeding in our experimental settings. Under thermoneutrality, the elevation of glucose concentrations in the drinking water resulted in accelerated tumour growth rates (Extended Data Fig. 6a). A concentration of 15% glucose resulted in the maximal effect of accelerated tumour growth, and further increases beyond this concentration did not additively affect tumour growth. On the basis of these findings, we decided to use 15% glucose in the drinking water for our subsequent experiments.

**Fig. 5:**
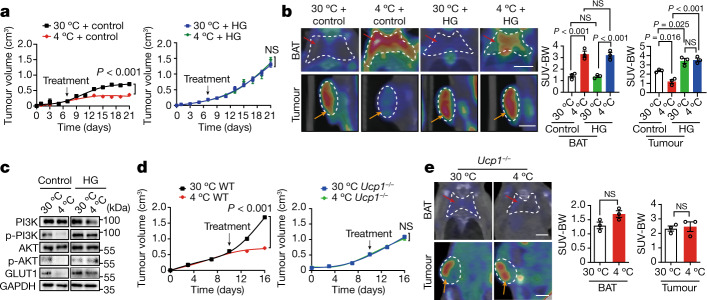
High-glucose feeding and UCP1 deficiency abrogate cold-induced tumour suppression. **a**, Tumour growth rates of CRC-tumour-bearing mice treated with control vehicle (left) or 15% glucose (right) under 30 °C and 4 °C. *n* = 5 mice per group. **b**, Representative PET–CT images and quantification of SUV-BW of tumour-bearing mice with equal tumour size treated with vehicle or 15% glucose under 30 °C and 4 °C. *n* = 3 mice per group. Scale bars, 5 mm. **c**, Western blotting detection of non-phosphorylated and phosphorylated PI3K, AKT and GLUT1 of CRC treated with vehicle or 15% glucose under 30 °C or 4 °C. GAPDH was used for standardizing total protein loading levels. **d**, Tumour growth rates of CRC implanted in WT (left) and *Ucp1*^−/−^ (right) mice under 30 °C and 4 °C conditions. *n* = 5 mice per group. **e**, PET–CT images of ^18^F-FDG uptake in the BAT and tumours of *Ucp1*^−/−^ CRC-tumour-bearing mice under 30 °C and 4 °C conditions. Red arrows indicate BAT and orange arrows indicate tumours. Quantification of SUV-BW values of BAT and tumours. *n* = 3 mice per group. Scale bars, 5 mm. For **a**, **b**, **d** and **e**, data are mean ± s.e.m. Statistical analysis was performed using one-way ANOVA followed by Tukey multiple-comparison tests (**b**) or two-sided unpaired *t*-tests (**a**, **d** and **e**). HG, high glucose (15% glucose). Source data

Under non-fasting and thermoneutral conditions, feeding mice with 15% glucose increased the level of blood glucose, that is, hyperglycaemia (Extended Data Fig. 6b). However, feeding mice with high glucose did not induce hyperglycaemia under a fasting condition (Extended Data Fig. 6b). Interestingly, feeding CRC-tumour-bearing mice with 15% glucose abolished the tumour inhibitory effect by cold exposure (Fig. 5a). Tumour cell proliferation, tumour hypoxia, microvessel density and inflammation remained unchanged in the 30 °C and 4 °C groups when feeding with 15% glucose (Extended Data Fig. 6c,d). Restoration of tumour growth by high-glucose feeding under cold exposure was reproduced in the melanoma and pancreatic ductal adenocarcinoma models (Extended Data Fig. 6e–j), further corroborating the high-glucose rescue effect. Consistently, metabolomics data showed that high-glucose feeding largely restored tumour glycolysis under cold exposure (Extended Data Fig. 6k,l). These findings demonstrate that compensation of blood glucose by high-glucose feeding obliterates cold-induced tumour suppression, suggesting that increased glucose uptake in activated BAT is an important mechanism for tumour suppression.

To further elucidate possible mechanisms underlying high-glucose feeding in the restoration of tumour growth, we analysed GLUT1 expression in CRC tumours under cold exposure. Interestingly, high-glucose feeding restored glucose uptake at 4 °C to a level that was indistinguishable from the 30 °C-high-glucose feeding group (Fig. 5b). Consistently, *Glut1* mRNA and protein expression levels during cold exposure were also elevated by high-glucose feeding (Extended Data Fig. 6m). Notably, high-glucose feeding significantly augmented the activation of PI3K and AKT by phosphorylation, and GLUT1 expression in cold-exposed tumours (Fig. 5c). These data suggest that high-glucose feeding restores glucose uptake through the GLUT1-mediated pathway.

## *Ucp1* deletion ablates tumour suppression

**Extended Data Fig. 7:**
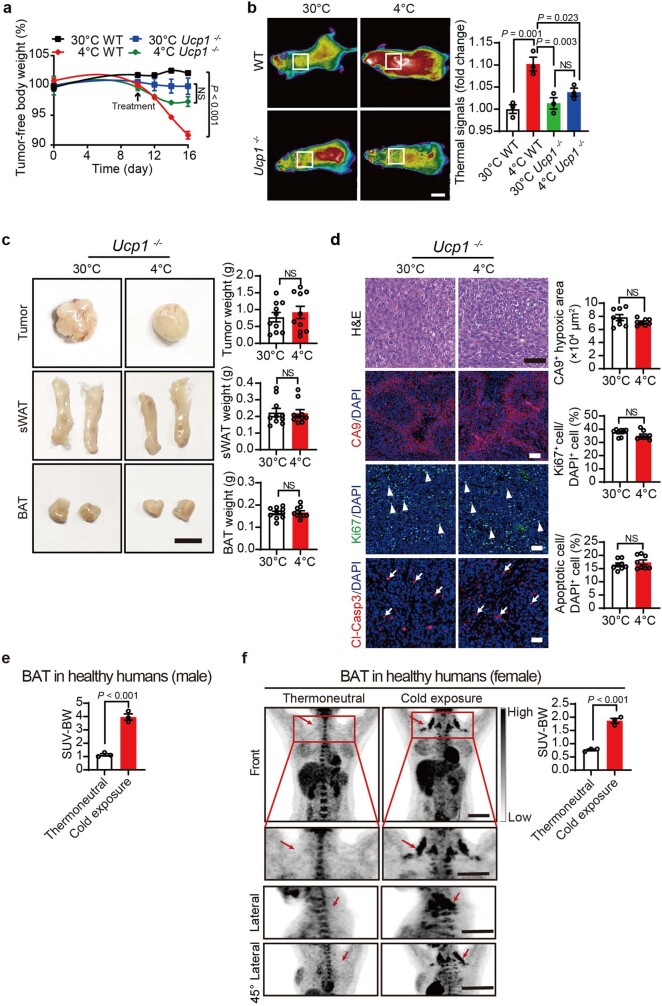
The impacts of UCP1 deletion in tumour microenvironment and the activation of BAT in healthy humans. **a**, Tumour-free bodyweights of CRC tumour-bearing WT and *Ucp1*^−/−^ mice under 30 °C and 4 °C (*n* = 6 mice per group). **b**, Images of infrared thermography from anaesthetized WT and *Ucp1*^−/−^ mice under 30 °C and 4 °C. Box points to BAT approximate location. Scale bar, 1 cm. Quantification of interscapular thermal signals of WT and *Ucp1*^−/−^ mice under 30 °C and 4 °C (*n* = 3 mice per group). **c**, Morphology and weight of CRC tumours, sWAT, and BAT of tumour-bearing *Ucp1*^−/−^ mice under 30 °C or 4 °C (*n* = 10 samples per group). Scale bar, 0.5 cm. **d**, Histological staining, immunofluorescence staining, and quantifications of CA9^+^ hypoxic area, Ki-67^+^ proliferating cells, and Cl-Casp3^+^ apoptotic cells in CRC tumour-bearing *Ucp1*^−/−^ mice under 30 °C and 4 °C (*n* = 8 random fields per group). Arrows and arrowheads point to positive signals. Scale bars, 50 μm. **e**, Quantification of ^18^F-FDG uptake in healthy male volunteers under thermoneutral (28 °C) and mild cold (16 °C) exposure for 2 weeks (*n* = 3 individuals). **f**, Representative PET images of ^18^F-FDG uptake by PET in healthy female volunteers under thermoneutral (28 °C) and mild cold (16 °C) exposure for 2 weeks. Scale bars, 100 mm. Quantification of SUV-BW positive signals presented in panel **f** (*n* = 3 individuals). Data presented as mean ± s.e.m. Statistical analysis was performed using one-way ANOVA followed by Tukey multiple tests (**a**, **b**) or two-sided unpaired *t*-test (**c**-**f**). NS, not significant. Source data

As UCP1 is the crucial mitochondrial protein responsible for NST in adipose tissues, we used *Ucp1*^−/−^ mice in our tumour experiments. In wild-type (WT) mice, cold exposure significantly reduced mouse body weight (Extended Data Fig. 7a). As expected, deletion of the *Ucp1* gene abolished this effect. Genetic deletion of *Ucp1* abolished the tumour-suppressive effect of cold exposure (Fig. 5d). Consistent with this observation, UCP1 removal markedly reduced heat production under cold acclimatization (Extended Data Fig. 7b). A PET–CT scan analysis showed non-significant glucose uptake in cold-exposed BAT (Fig. 5e). However, the tumour tissue in *Ucp1*^−/−^ tumour-bearing mice exhibited unaltered glucose uptake relative to the tumours grown in WT mice (Fig. 5e). Tumour, BAT and sWAT sizes in the 4 °C group of *Ucp1*^−/−^ mice were similar to those in the 30 °C group (Extended Data Fig. 7c). Similarly, deletion of *Ucp1* elevated tumour cell proliferation and hypoxia to the thermoneutral levels (Extended Data Fig. 7d). Tumour glycolysis analysed by metabolomics was largely restored in *Ucp1*^−/−^ mice (Extended Data Fig. 6k,l). These findings support the fact that the UCP1-mediated mechanism is essential for tumour suppression.

## BAT activation in a human patient with cancer

**Fig. 6:**
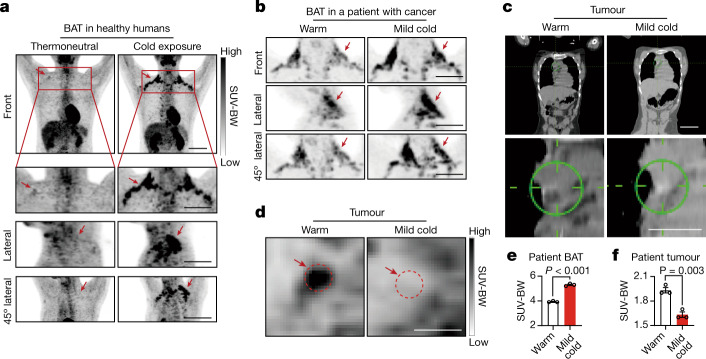
Activation of BAT by exposure to mild cold in healthy individuals and a patient with cancer. **a**, Representative PET images of ^18^F-FDG uptake in healthy male volunteers under thermoneutral (28 °C) and cold exposure (16 °C) conditions for 2 weeks. *n* = 3 individuals. Scale bars, 100 mm. **b**, Representative PET images of ^18^F-FDG uptake in the bilateral areas of supraclavicular, cervical and parasternal regions in a patient with Hodgkin’s lymphoma under warm and mild cold conditions. Scale bars, 100 mm. **c**, Representative images by CT scanning in the tumour region under warm conditions and exposure to mild cold. Scale bars, 100 mm (top) and 50 mm (bottom). **d**, PET images of ^18^F-FDG uptake of the tumour region under warm and mild cold conditions. Scale bars, 50 mm. Arrows indicate respective positive signals. **e**, Quantification of SUV-BW positive signals shown in **b**. *n* = 3 separate scans. **f**, Quantification of SUV-BW positive signals shown in **d**. *n* = 3 separate scans. For **e** and **f**, data are mean ± s.e.m. Statistical analysis was performed using two-sided unpaired *t*-tests (**e** and **f**). Source data

To study the human relevance of our findings in various animal models, we performed tolerable cold exposure in healthy humans and in a patient with cancer. Healthy volunteers (3 males and 3 females aged between 22 and 25 years) were recruited for the study. Wearing very light clothing, these healthy individuals were exposed to a mildly cold ambient temperature at 16 °C for 2–6 h per day for a consecutive 14 days. PET scan analysis showed that several individuals from both the male and female groups exhibited marked activation of BAT in the bilateral areas of supraclavicular, cervical and parasternal regions (Fig. 6a and Extended Data Fig. 7e,f). The degree of BAT activation varied substantially between individuals and typical examples of ^18^F-FDG uptake demonstrated that a significant amount of BAT existed in adult male and female individuals, and it was activated by tolerable cold exposure (Fig. 6a and Extended Data Fig. 7e,f).

We next performed a pilot study on an 18-year-old patient with Hodgkin’s lymphoma. At the time of this study, this patient had received a combination chemotherapy of adriamycin, bleomycin, vinblastine and dacarbazine for 5 cycles. Our study was performed at the fifth cycle of chemotherapy intervals. This patient, wearing light clothing, was exposed to mild cold of 22 °C for 7 days. PET–CT scanning demonstrated significant amounts of BAT in the bilateral areas of supraclavicular, cervical and parasternal regions and became activated with heavy uptake of ^18^F-FDG (Fig. 6b). However, exposure of this patient to a warm 28 °C ambient environment for 4 days markedly reduced ^18^F-FDG uptake and BAT activation (Fig. 6b). The CT-imaging analysis confirmed the existence of lymphoma in the mediastinal area (Fig. 6c), which demonstrated markedly mitigated ^18^F-FDG uptake during warm exposure (Fig. 6d–f). These preliminary and pilot findings demonstrate that (1) a significant amount of BAT exists in adult humans; (2) after mild tolerable cold exposure, BAT became activated confirmed by ^18^F-FDG uptake; (3) BAT was also activated by cold exposure in a patient with cancer; and (4) exposure of a patient with cancer to mild cold conditions markedly reduced glucose uptake in the tumour tissue. Together, these human data firmly link our preclinical findings to clinical relevance, although more rigorous clinical studies are needed to strengthen these conclusions.

## Discussion

Cancer cells signify a metabolic machinery in which exchanges of biomolecules of sugars, lipids, amino acids, metabolites and gases vigorously occur through distinct metabolic pathways to facilitate tumour growth and metastasis. Among these metabolic pathways, cancer glycolysis—that is, the Warburg effect—is probably the best-characterized mechanism for supporting tumour growth by generating ATP as energy molecules^1,13^. Accumulating experimental evidence shows that genetic and pharmacological interference of the glycolytic pathway markedly attenuates cancer cell proliferation and tumour growth^42,43^. On the basis of this principle, therapeutic agents targeting aerobic glycolysis have been developed and entered clinical trials for treating patients with cancer^44^. Here we provide a therapeutic concept for cancer treatment by switching on thermogenic metabolism in BAT. Notably, exposure of tumour-bearing mice to cold results in potent inhibitory effects on tumour growth and the cold-induced antitumor activity is at least equivalently potent to most anticancer drugs. Exposure of tumour-bearing mice to mild cold 22 °C did not result in tumour suppression. Perhaps, 22 °C was not sufficiently low to fully activate BAT in our experimental models. It has been described that a few degrees difference of ambient temperature can affect tumour growth^45^.

This therapeutic approach is simple, cost-effective and feasible in almost all hospitals and even at home, and is most likely omnipresent for all cancer types. As adult humans have a sufficient amount of BAT that effectively takes up blood glucose after cold exposure, we reasonably speculate that a similar anticancer activity should also be seen in human patients with cancer. Our preliminary results corroborated previous findings that adult healthy male and female individuals possess a substantial amount of BAT in the supraclavicular, cervical and parasternal regions, which was activated by exposure to mild cold. The short exposure to mild cold ambient temperature of 16 °C is well tolerated by adult humans without causing any adverse effects. Marked decreases in ^18^F-FDG uptake in the tumour tissue and simultaneous activation of BAT in a patient with cancer validate the concept of jeopardizing cancer glycolysis by cold exposure. Although it is difficult to draw conclusions from data obtained from a single patient with cancer, these preliminary findings at least demonstrate the clinical relevance of our study. Rigorously designed clinical studies are warranted for strengthening this concept. Together, our findings represent a new concept for cancer therapy that patients with cancer could potentially benefit from.

We undertook three experimental approaches, including: (1) measuring subcutaneous temperature by a thermal probe; (2) implantation of tumours in internal organs; and (3) spontaneous tumours in genetic mouse models to exclude the possibility that direct contact of a subcutaneous tumour with low temperature deterred tumour growth. Under cold exposure, the subcutaneous temperature slightly increased rather than decreased, probably due to the robust NST in the animal body. Similarly, CBT remains unaltered during cold exposure relative to thermoneutrality. Consistent with the overall evidence of thermogenic alterations, tumours implanted into internal organs, such as the liver, also show impaired growth rates. Moreover, suppression of intestinal adenoma formation and growth in the genetic *Apc*^*Min/+*^ model further excludes the anticancer effect by chilling the tumour tissue. These experimental settings provide compelling evidence to exclude the likelihood that the attenuated tumour growth rates were caused by low temperature alone. Along this line of interpretation, surgical removal of BAT reverses the cold-associated reduction in tumour growth rates, providing powerful evidence of BAT-activation-manipulated tumour growth. Similarly, genetic deletion of *Ucp1* also ablates cold-induced tumour suppression. However, genetic deletion of *Ucp1* has been shown to result in mitochondrial deficiency in mice^46^. The UCP1-dependent tumour suppression by cold may potentially reflect the mitochondrion-dependent suppression by BAT.

The advantages of using spontaneous tumour models of genetically propagated mouse strains encompass: (1) excluding the possibility that suppressing tumour growth by cold occurs only in fast-growing xenograft tumours, which are highly dependent on glycolysis. (2) Establishing the clinical relevance of our findings. (3) Testing the possibility of cold-triggered tumour formation. In the *Apc*^*Min/+*^ model, we showed that chronic cold exposure also potently suppressed tumour formation in addition to inhibition of tumour growth. Finally, (4) studying the effect of chronic exposure to cold on tumour suppression. These results demonstrate that activation of BAT thermogenesis is responsible for tumour inhibition.

The ^18^F-FDG PET–CT scanning analysis demonstrates that BAT, but not browning WAT, is the key organ responsible for glucose uptake. This finding suggests that accelerated glucose uptake by cold-activated BAT is probably the key mechanism underlying tumour suppression. However, we cannot exclude the participation of WAT, especially sWAT, in tumour suppression by cold. A recent study shows that a subpopulation of beige cells in sWAT is almost completely dependent on glucose uptake^47^. Perhaps extreme cold is required for triggering the WAT-mediated tumour suppression because extreme cold augments robust browning in WATs^48^. In addition to increases in glucose uptake, thermogenic adipose tissues also exhibit increased uptake of other nutrients and regulatory molecules for tumour growth, including succinate and branched-chain amino acids^49,50^, which may also participate in tumour suppression by cold exposure.

**Extended Data Fig. 8:**
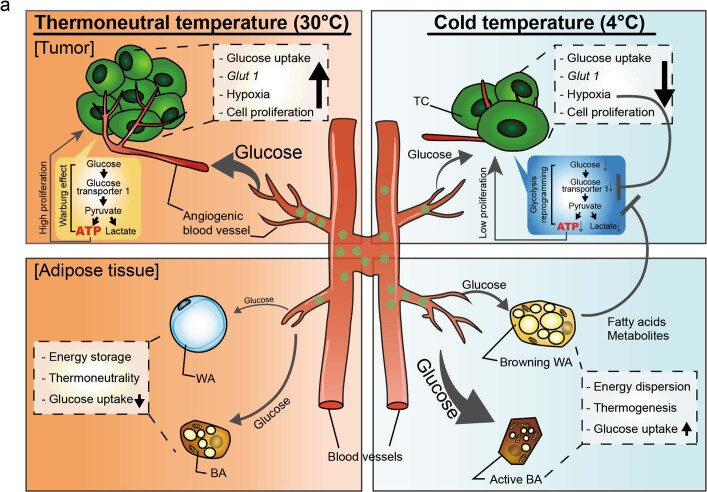
Schematic diagram of mechanisms underlying tumour suppression by cold exposure. Under the thermoneutral temperature, cancer cells mainly acquire their energy through glycolysis, that is, the Warburg effect. Angiogenic vessels in tumours provide sufficient glucose for tumour consumption. Both WAT adipocytes (WAs) and BAT adipocytes (BAs) remain metabolically inert without significant glucose uptake and thermogenic activity. Glycolysis-generated ATP molecules and other metabolites support tumour cell proliferation at high rates. Under cold exposure, both sWAT and BAT undergo a browning process that markedly increases glucose uptake and thermogenesis. Owing to the elevated glucose uptake in activated BAT and sWAT, glucose uptake in tumours is significantly reduced. Consequently, tumour growth under cold exposure is inhibited through several possible mechanisms: 1) reduced glucose supply in tumours due to glucose redistribution in activated BAT and WAT; 2) mitigation of tumour hypoxia. Owing to limited glycolysis, acidosis and tumour hypoxia are reduced. Additionally, slow tumour growth also alleviates tumour hypoxia. Mitigation of tumour hypoxia by cold exposure also reduces expression levels of GLUTs, which are limiting factors for glucose uptake; and 3) plausible inhibition of glycolysis by lipid metabolites.

There are several plausible mechanisms that underlie BAT-activation-triggered tumour suppression (Extended Data Fig. 8a): (1) deprivation of ATP production in cancer cells by limiting glucose supply. (2) Production of intermediary metabolic products that suppress the Warburg effects and growth of cancer cells. (3) Reduction of tumour-growth-stimulating factors and cytokines. BAT activation and WAT browning are known to reduce inflammation, which is one of the hallmarks of cancer. Finally, (4) restriction of lipid supply due to active lipolysis. The division of cancer cells constantly demands a lipid supply for constructing the plasma membrane and organelle membranes, and thermogenic metabolism competes for lipid consumption. Among these possibilities, limiting glucose supply is probably the most important mechanism for tumour suppression because feeding tumour-bearing mice with high-glucose supplements largely reinstates tumour growth rates.

Our findings provide an example of how alteration of global metabolism substantially affects tumour growth. This concept can be generalized in other global metabolic states, which may positively or negatively affect tumour growth and even metastasis. Opposing our results, overweight and obesity accelerate tumour growth, metastasis and drug responses by providing excessive energy for cancer cells^31–33^. One of the key findings of our study is the restoration of tumour growth under cold exposure by high-glucose feeding. If GLUT1 reduction is the limiting step for glucose uptake in tumour cells, feeding tumour-bearing mice with a high-glucose diet may not be able to recuse tumour growth. Thus, additional mechanisms are probably entailed in restoration by hyperglycaemia. We provide evidence showing that high-glucose feeding restores glucose uptake, GLUT1 expression, and AKT and PI3K activation in tumours during cold exposure. A possible mechanistic link between high-glucose feeding and accelerated tumour growth may involve the high-glucose–GLUT1 signalling axis. It is also probable that high-glucose feeding also increases expression levels of insulin and insulin-like growth factor (IGF), which may secondarily affect GLUT1 expression. The in-depth mechanism warrants further investigation.

Taken together, we show that the activation of BAT by exposure to physiologically tolerable low temperatures provides an effective approach for cancer therapy. The therapeutic effectiveness of cold exposure is at least equivalent to most available anticancer drugs.

## Methods

### Cell culture

The mouse MC-38 colon adenocarcinoma cell line was provided by R. Hernández. The mouse E0771 breast cancer cell line was purchased from CH3 BioSystems. The mouse pancreatic cancer cell line Panc02 was provided by M. Schnurr. The human colorectal carcinoma HCT 116 tumour cells, human pancreas duct epithelioid carcinoma PANC-1, mouse fibrosarcoma T241 and mouse melanoma B16-F10 were purchased from ATCC. T241, HCT 116, Panc02, PANC-1 and B16-F10 cells were cultured and maintained in a Dulbecco modified Eagle's medium (DMEM, D6429, Merck) supplemented with 10% FBS, 100 U ml^−1^ penicillin and 100 μg ml^−1^ streptomycin (P4333, Merck). MC-38 cells were cultured and maintained in DMEM supplemented with 10% FBS, 10 mM HEPES, 0.1 mM MEM non-essential amino acid (M7145, Merck), 100 U ml^−1^ penicillin and 100 μg ml^−1^ streptomycin. E0771 cells were cultured and maintained in Roswell Park Memorial Institute (RPMI) 1640 (SH30027.01, GE Healthcare Life Science) medium supplemented with 10% FBS, 10 mM HEPES, 100 U ml^−1^ penicillin and 100 μg ml^−1^ streptomycin. All of the cell lines used in our study were negative for mycoplasma as detected by a microplasma kit (LT07-318, Lonza).

### Animals

C57Bl/6 and SCID mice were obtained from the breeding unit at the Department of Microbiology, Tumour and Cell Biology, Karolinska Institute, Stockholm, Sweden and from the Model Animal Research Center of Nanjing University. *Ucp1*^−/−^ mice in the C57BL/6 background were purchased from the Jackson Laboratory (003124). Female 6–8-week-old FVB/NJGpt-Tg(MMTV-PyMT)/Gpt mice were purchased from GemPharmatech (T004993). Male 9–10-week-old C57Bl/6J background *Apc*^*Min/+*^ mice (002020) were obtained from the Jackson Laboratory. Adult males and female mice aged 5–12 weeks were caged at 22 ± 3 °C under 12 h–12 h light–dark cycles before cold and warm exposure. Mice were randomly divided into groups for all of the experiments. All of the mouse studies were approved by the North Stockholm Animal Ethical Committee, Stockholm, Sweden (N3/17 and 6196-2019 with a maximal tumour volume of 2.5 cm^3^ and 2.0 cm^3^ for subcutaneous tumours and intraorgan tumours, respectively), or by the Animal Experimental Ethical Committee of the Fudan University, Shanghai, China (20190430 with a maximal tumour volume of 3.0 cm^3^). No statistical methods were used to predetermine sample size. The experimenter was not blinded to the assignment of the groups and the evaluation of the results. Tumour experiments, including subcutaneous, intraorgan implantation and genetic tumour models were performed at the Karolinska Institutet, Sweden and Fudan University, China. In both locations, subcutaneous tumour volumes did not exceed 2.5 cm^3^ and intraorgan tumour volumes did not exceed 2.0 cm^3^ in any of the experiments.

### Cold exposure

For cold exposure, mice were housed and placed in an opened cage in the climate room at the Department of Microbiology, Tumour and Cell Biology, Karolinska Institute, Stockholm, Sweden, at the Department of Cellular and Genetic Medicine, School of Basic Medical Sciences, Fudan University, or in a climate chamber (HPP750, Memmert or HWS-350FT, Binglin Electronics). Male and female C57Bl/6 mice aged between 5 and 10 weeks were adapted at 18 °C for 1 week before exposing to a cold temperature of 4 °C in the climate room or 5 °C in the HPP750 climate chamber or exposed to a thermoneutral temperature of 30 °C as a control, followed by tumour implantation.

### Mouse tumour models

Approximately 1 × 10^6^ cells of T241 fibrosarcoma, E0771 breast cancer and B16 melanoma, 1–3 × 10^6^ MC-38 tumour cells, 1.5 × 10^6^ Panc02 cells and 5 × 10^6^ PANC-1 cells in 100 μl phosphate-buffered saline (PBS) were subcutaneously implanted into each of C57BL/6 mouse, or immune-deficient SCID mice. For the intraliver injection model, in isoflurane-anaesthetized 7-week-old female C57BL/6 and 10-week-old male SCID mice, a subcutaneous incision was performed and the mice were injected with approximately 0.5 × 10^6^ MC-38 or 1 × 10^6^ HCT 116 cells in 30 μl PBS into the liver, followed by closure of the incisions with the sterile surgical suture (CR436, Jinhuan Medical). All surgically operated mice were subcutaneously treated with buprenorphine before and after surgical operation. Tumour sizes were measured with callipers and tumour volumes were calculated according to the standard formula (length × width^2^ × 0.52). For the subcutaneous tumour model, tumour-bearing mice were euthanized when the tumour size reached the indicated size and tissues including tumours, WAT depots, iBAT and liver were dissected for further assessments. Fresh tumour, WAT and BAT were immediately collected and kept on dry ice or liquid nitrogen for metabolomics and RNA-seq multi-omics analyses. Mice in the intraliver tumour models were euthanized 2–3 weeks or 6 weeks after tumour implantation. For RNA and protein extraction, fresh tissues were immediately frozen on dry ice and stored at –80 °C until further use. A portion of fresh tissues was immediately dissected, fixed with 4% paraformaldehyde (PFA) (MA0192, Meilunbio) overnight and subsequently used for histological and immunohistochemical analyses. Metabolic rates were measured when tumours reached an average size of approximately 1.0 cm^3^. Mice were analysed for metabolic rates, followed by euthanasia and tissue collection. Vehicle-treated mice using the same therapeutic regimens served as a control group.

Female 10-week-old MMTV-PyMT mice were adapted at 18 °C for 24 h before exposing to 4 °C or 30 °C conditions. MMTV-PyMT mice started to develop tumours in the mammary glands at the age of 12 weeks. For BAT-removal experiments, BAT of each animal was removed at the age of 9 weeks, cold exposure started at the age of 10 weeks and continued until the termination of the experiments. At week 4 after cold exposure, PET–CT analysis was performed and tumour nodules of the same size from the 4 °C- and 30 °C-exposed groups were analysed for ^18^F-FDG uptake. C57Bl/6J background *Apc*^*Min/+*^ male mice (aged 9 weeks) were acclimatized to 4 °C and 30 °C for 6 weeks. *Apc*^*Min/+*^ mice usually developed multiple intestinal adenomas between weeks 10 and 14. On week 6 after cold exposure, PET–CT analysis was performed and tumour nodules of the same size from the 4 °C- and 30 °C-exposed groups were analysed for ^18^F-FDG uptake.

### Body and intratumour temperature

To measure mouse CBT, a temperature probe (TW2 and RET-3, Agntho’s AB) was placed in the rectum of each mouse. For measuring subcutaneous body temperature, a small incision on the interscapular region of each mouse under mild isoflurane anaesthesia was surgically created and the probe was subsequently inserted into the incision for measurements. Accurate temperature was read using the MicroTherma 2T thermometer. For measuring tumour temperature, the probe was put into the tumour of the mice. All measurements were performed at room temperature.

### Metabolic analyses

Whole-body energy metabolism and non-shivering thermogenesis were quantitatively measured by oxygen consumption using the Oxymax CLAMS-HC comprehensive laboratory animal monitoring system with environmental enclosure (Columbus Instruments). The metabolic measurements were carried out at thermoneutral and cold temperatures. The oxygen sensor was warmed up for at least 6 h before calibration with the reference gases, that is, 100% nitrogen gas and a mixture of 20.5% O_2_ and 0.5% CO_2_. The environmental enclosure was warmed and cooled before placing the mice inside. For the whole-body energy metabolism, CO_2_ and O_2_ were measured and the data were collected every 12 min for each experimental mouse.

### Glucose- and insulin-tolerance tests

Mice were starved for 4–6 h during the light phase with free access to water on the day of assessment. Glucose levels in the blood samples collected from the tail vein by a 25G needle were measured using a glucometer (Accu-Chek, Active, Roche Diagnostics) immediately before and at 15, 30, 60 and 120 min after oral feeding with 1.5 mg glucose with 10 μl per g body weight and intraperitoneal injection of insulin (0.5 U per kg body weight), respectively. Mice were kept at ambient temperature throughout these assays.

### PET–CT imaging

Mice that were exposed to 4 °C or 30 °C were fasted for 6 h before PET–CT scanning. ^18^F-fluoro-2-deoxy-d-glucose (^18^F-FDG) with a radiochemical purity of more than 95% was produced by a cyclotron (Siemens CTI RDS Eclips ST) using the Explora FDG4 module. In vivo PET–CT imaging scans and image analysis were performed using an Inveon Animal-PET-CT system (Siemens Preclinical Solution). Mice exposed to 4 °C or 30 °C were scanned 1 h after intravenous injection of 3.7 MBq (100 μCi) of ^18^F-FDG. Experimental animals were maintained with anaesthetization using 2.5% isoflurane/oxygen before and during the scanning. Three-dimensional ordered-subset expectation maximization (3D-OSEM)/maximum algorithm was used for image reconstruction. The maximal percentage-injected dose per gram was calculated and the SUV-BW of iBAT and tumour in either the subcutaneous region or liver was measured in a manually drawn region of interest. Inveon Acquisition Workplace software (Siemens Medical Solutions) was used for further analysis.

For human studies, CT scans were performed using the following protocols: 120 kv tube voltage, 120 mA tube current and 3.75 mm thickness of layers. For PET collection, 6–7-bed cycles were used for the whole-body scanning and each bed cycle was 2 min. Data were reconstructed using the viewpoint method.

### Surgical removal of iBAT

For removal of iBAT, a small incision was surgically created in each mouse under isoflurane-inhalation anaesthetization. Blood vessels in the iBAT were crushed by a pinch using sterilized forceps to prevent excess bleeding after cutting out the tissues. iBAT tissues were carefully dissected using autoclaved surgical scissors, followed by the closure of the incision with the sterile surgical suture. Sham-operated mice were used as controls. All of the mice were allowed to recover for a week at ambient temperature before further analyses.

### Survival assay

Survival studies of tumour-bearing mice were performed at Fudan University, Shanghai, China according to the ethical permit in which the humane end point (tumour size ≤ 3 cm^3^) was the criterium to euthanize each mouse. This end point and body condition score BCS-1 (moribund state) were not exceeded in any of the experiments.

### RNA-seq analysis

For RNA-seq, isolated total RNA was collected from isolated tumour tissues and was measured using the NanoDrop One Microvolume UV-Vis Spectrophotometer (Thermo Fisher Scientific). The quality of RNA samples was assessed by agarose gel electrophoresis and using the Agilent 2100 Bioanalyzer (Agilent Technologies). All of the samples displayed a 260/280 ratio of around 2.0. cDNA libraries were constructed and sequenced by Majorbio Biotech. In brief, 200 ng RNA for each group was used for the library construction using the Truseq RNA sample prep Kit (Illumina). The constructed DNA was enriched by PCR amplification and then purified by Certified Low Range Ultra Agarose (Bio-Rad) gel electrophoresis. Clone clusters were generated on the Illumina cBot, using the Truseq PE Cluster Kit v3-cBot-HS, and high-throughput sequencing was performed on an Illumina Miseq sequencer, using Truseq SBS Kit v3-HS (200 cycles). Aligned reads were summarized using featureCounts (v.2.0.0) based on the Gencode vM19 annotation. Differential expression analysis between three 30 °C and two 4 °C samples was performed using the R (v.4.0.3) package DESeq2 (v.1.30.0). GSEA was performed with GSEA (v.4.1.0) using the GSEAPreranked tool, whereby genes were preranked on the basis of their *P* values and fold changes. Mouse gene sets for Gene Ontology analysis were downloaded from http://ge-lab.org/gskb/.

### Metabolomics analysis

Metabolomics analysis was performed in house or with the assistance from Shanghai Biotree Biotech. Metabolites in MC-38 tumours and adipose tissues were measured by capillary electrophoresis time of flight mass spectrometry (CE–TOFMS) using the Agilent 7100 CE Capillary Electrophoresis system equipped with an Agilent 6230 Time-of-Flight mass spectrometer (Agilent Technologies) according to previously reported methods. In brief, snap-frozen tumour and adipose tissue samples (approximately 100 mg for tumour and 30 mg for adipose tissues) were homogenized with 500 μl of methanol containing internal standards (H3304-1002, Human Metabolome Technology) and 500 μl of chloroform and 200 μl of ultrapure water (50% v/v) were subsequently added into the sample solution. The solution was centrifuged at 2,300*g* for 5 min at 4 °C to remove debris. Subsequently, 300 μl of the aqueous fraction was centrifugally filtered through a 5 kDa cut-off filter using an Ultrafree MC-PLHCC filter (UFC3LCCNB_HMT, Human Metabolome Technology). The filtrate was dried by a dryer for 180 min and dissolved in 25 μl of both Milli-Q water-containing reference compounds (H3304-1004, Human Metabolome Technology). The amount of all anion- and cation-charged metabolites in the samples was measured by CE−TOFMS. For analysis of cationic metabolites, a fused silica capillary (H3305-1002, 50 μm internal diameter × 80 cm total length, Human Metabolome Technologies) with a cation buffer solution (H3301-1001, Human Metabolome Technologies) as the electrolyte was used. The sample was injected at a pressure of 50 mbar for 10s. To determine anionic metabolites, a chemically coated cationic polymer capillary (50 µm internal diameter × 80 cm total length, H3305-1002, Human Metabolome Technologies) was used with an anion buffer solution (H3302-1021, Human Metabolome Technology) as the electrolyte. The sample was injected at a pressure of 50 mbar for 25 s. For measuring cation- and anion-charged metabolites, the CE capillary was preconditioned by flushing the running electrolyte for 3 or 4 min before sample injection. Each sample was inserted with a pressure injection of 50 mbar for 10 s under 20 °C of the capillary temperature and 27 kV and 30 kV applied voltage for measurement of cation- and anion-charged metabolites, respectively. Sheath liquid (H3301-1020, Human Metabolome Technology) was delivered at 10 µl min^−1^ to the CE interface by a pump using the Agilent 1200 series pump equipped with a sheath flow splitter. Electrospray ionization–TOFMS was conducted in positive-ion mode with 4 kV capillary voltages for cationic metabolites and was set in the negative-ion mode with 3.5 kV for anionic metabolites.

The spectrometer was scanned from *m*/*z* 50 to 1,000 at 1.5 cycles per second during separation and detection. An automatic recalibration of each acquired spectrum was performed using the masses of reference standards. For the CE–TOFMS system control and data acquisition, we used an Agilent MassHunter software for TOFMS (Agilent Technologies). All target metabolites were identified by matching their *m*/*z* values and migration times with the normalized *m*/*z* values and migration times of corresponding authentic standard compounds. Data were quantified by comparing the ratio of analyte peak area/internal standard peak area of sample solution to that of the reference standard mixture. The quantitative formula was as follows: *X* nmol mg^−1^ = (sample relative area)/(standard relative area) × (concentration of internal standard in methanol: 50 µM) × (amount of methanol for metabolite isolation (500 µl)) × 1/(sample weight) × (concentration of metabolite in the reference standard mixture (50 µM))/(concentration of internal standard in the reference standard mixture (100 µM)). The sample relative area = (metabolite peak area in the sample solution)/(internal standard area in the sample solution). The standard relative area = (metabolite peak area in the reference standard mixture)/(internal standard area in the reference standard mixture). Chromatograms of each metabolite were extracted and quantified using the Agilent MassHunter Qualitative Analysis software (Agilent Technologies). For lipidomics analysis, 50 μl mouse serum sample was diluted into extraction buffer (isopropyl alcohol (CAEQ-4-013493-4000, CNW Technologies): *n*-hexane (CAEQ-4-011518-4000, CNW Technologies) = 2:3 (v:v) with internal standards (CDAA252795, ANPEL). Samples were sonicated in an ice-water bath for 5 min, then centrifuged at 4 °C for 15 min at 12,000 rpm. Then, 400 μl of supernatant was dried in a speed vac, stored under nitrogen and reconstituted in 160 μl of *n*-hexane. After centrifugation at 12,000 rpm for 5 min, the supernatant was projected for gas chromatography–mass spectrometry (5977B, Agilent) for further analysis.

### Drug treatment

CL-316,243 disodium salt (1499, Tocris Biosciences) was administered systemically by daily intraperitoneal injection at a dose of 1 mg kg^−1^ in PBS. After 21 days of treatment, the mice were euthanized for further analysis.

### Infrared thermal imaging

Mice in thermoneutral and cold environments were anaesthetized and kept at room temperature for 5 min for imaging. Thermal images on the back of mice were captured using an infrared thermal imager (Fortric 285, Fortric). Setting environmental temperature as the baseline, thermal images of mice were acquired and were further analysed using Fotric AnalyzIR.

### Histology and immunohistochemistry

The paraffin-embedded tissue sections of 5 μm thickness were incubated at 60 °C for approximately 2 h to melt the covered paraffin. After the incubation, the warm slides were put in Tissue-Clear (1466, Sakura) for deparaffinization and dehydrated in serial steps using 99%, 95% and 70% ethanol. For haematoxylin and eosin (H&E) staining, the dehydrated slides were stained with H&E. For immunohistochemical staining, tissue slides were boiled by microwave for 20 min in an unmasking solution (H3300, Vector Laboratories) and subsequently blocked with 4% either goat or donkey serum. To visualize cells in the adipose tissues and TME, we stained the WAT, BAT and tumours with antibodies listed below. For the TME, the slices of tumour tissue were stained with rabbit anti-mouse cleaved-caspase-3 (1:200, 9661, Cell Signaling), rabbit anti-mouse Ki-67 (1:100, PA5-19462, Thermo Fisher Scientific), rabbit anti-mouse FSP1 (1:300, 07–2274, Merck), rabbit anti-mouse IBA1 (1:200, 019–19741, FUJIFILM Wako) or rabbit anti-mouse CD45 (1:200, ab10558, Abcam) antibodies, followed by staining with species-matched secondary antibodies as follows: Alexa Fluor 555-labelled goat anti-rabbit (1:300, A21482, Thermo Fisher Scientific) or Alexa Fluor 488-labelled donkey anti-rabbit (1:300, A21206, Thermo Fisher Scientific) antibodies. For detection of hypoxia in the tumour, the slides were stained with a rabbit anti-CA9 antibodies (1:300, NB100–417, Novus) for primary staining and Alexa Fluor 488-labelled donkey anti-rabbit or Alexa Fluor 555-labelled goat anti-rabbit antibodies for the secondary staining. For adipose tissues, the slide with the samples was stained with rabbit anti-mouse UCP1 (1:200, ab 10983, Abcam), rabbit anti-mouse COX4 (1:300, GTX114330, GeneTex), and guinea pig anti-mouse perilipin (1:300, 20R-PP004, Fitzgerald Industries) antibodies, followed by dyeing with Alexa Fluor 555 goat anti-rabbit and Alexa Fluor 647 goat anti-guinea pig (1:200, A-21450, Thermo Fisher Scientific) secondary antibodies. Positive signals were detected using a fluorescence microscope equipped with a camera (Nikon, DS-QilMC) using NIS-Elements D3.2 (Nikon). For H&E staining, the image was captured using a light microscope (Nikon Eclipse TS100) with the camera (DS-Fil, Nikon) using NIS-Elements F v.3.0 software (Nikon). Images were analysed using Adobe Photoshop CS5 extended software and ImageJ.

### Whole-mount staining

Paraformaldehyde-fixed tumours, sWAT and iBAT tissue samples were digested with 20 μg ml^−1^ proteinase K (EO0491, Thermo Fisher Scientific) in a 10 mM Tris-HCl buffer (pH 7.4) for 5 min and blocked with 3% skim milk, followed by staining overnight at 4 °C with goat anti-mouse CD31 antibodies (1:200, AF3628, R&D systems). After rigorous rinsing with PBS, blood vessels were detected with donkey anti-goat Alexa Fluor 555-labelled secondary antibodies (1:300, A21432, Thermo Fisher Scientific), mounted in Vectashield mounting medium (Vector Laboratories), and stored at −20 °C in the dark before microscopy examination using the Nikon C1 confocal microscope (Nikon). The images were recorded using the camera of a Nikon C1 confocal microscope using the EZ-C1 v.3.91 software (Nikon). Captured images were further analysed using Adobe Photoshop CS5 extended software.

### FACS analysis

Approximately 3 × 10^6^ MC-38 GFP tumour cells in 100 μl PBS were subcutaneously implanted into each C57BL/6 mouse. Tumour tissues were collected around 0.5 cm^3^ in size, and the fresh tumour tissues were cut into small pieces, followed by collagenase digestion. In brief, the minced tumours were enzymatically digested with 0.15% collagenase I (17100-017, Gibco) and 0.15% collagenase II (17101015, Gibco) in PBS for 40 min at 37 °C, and centrifuged at 1,500 rpm at 4 °C for 10 min. The pellets were resuspended with PBS containing 1% FBS. Single-cell pellets were obtained by filtration using 70 μm cell strainers followed by centrifugation at 1,300 rpm at 4 °C for 10 min. The pellets were fixed using 1% PFA in PBS for 15 min at room temperature and washed in PBS. The cell pellets were further resuspended in PBS, and cold 70% ethanol was added dropwise to the pellets while vortexing. The samples were stored at 4 °C for further analysis. To the DNA content, fixed single-cell suspensions in PBS were incubated with 20 mg ml^−1^ propidium iodide (PI) (P3566, Invitrogen) and 100 mg ml^−1^ RNase (EN0531, Thermo Scientific) final on ice for 30 min. PI signals in GFP-positive cells were immediately analysed on the BD FACSCalibur Flow Cytometer (BD Bioscience), and data analysis was performed using the CellQuest Pro software (v.6.0, BD Bioscience). Gating strategies are shown in Supplementary Fig. 1. GFP-positive MC-38 cells and PI unstained samples were used to set each gate.

### RNA isolation and PCR analysis

Total RNA was extracted from cells and tissues using the TRIzol (15596026, Invitrogen) and GeneJET RNA Purification Kits (K0732, Thermo Fisher Scientific) according to the manufacturer’s instructions. The total RNA was reverse-transcribed and cDNAs were used for PCR and qPCR analyses using the primers listed in Supplementary Table 1. Samples were stored at −20 °C and processed for qPCR using the ABI Prism 7500 System (Applied Biosystems). Each qPCR sample was performed in triplicate and 20 µl reactions contained the Power SYBR Green PCR Master Mix (4367659, Thermo Fisher Scientific) or Hieff qPCR SYBR Green Master Mix (11203ES03, Yeasen), 150 nM forward and reverse primers and 1 µl cDNA. The qPCR protocol was performed for 40 cycles and each cycle consisted of denaturation at 95 °C for 15 s, annealing at 60 °C for 1 min, and extension at 72 °C for 1 min.

### LDH activity

Freshly isolated mouse CRC tumours were washed and immersed with the assay buffer, and then homogenized using a Dounce homogenizer. Tissue lysates were applied to the LDH activity assay using the LDH assay kit (ab102526, Abcam) according to the manufacturer’s instructions.

### Immunoblotting

Tissue lysates from tumours in different mouse models were extracted using a lysis buffer (C3228, Sigma-Aldrich) with a mixture of proteinase inhibitors (1:100, 8340, Sigma-Aldrich) and a phosphatase inhibitor cocktail (1:100, 5870, Cell Signaling) by a homogenizer (15555819, Thermo Scientific). Protein concentrations in tissue lysates were measured using the BCA assay. An equal amount of protein from each experimental group and a protein ladder (26616/26620, Thermo Scientific) was placed onto an SDS–PAGE gel (4561086/4561083, Bio-Rad) or a 10% SDS–PAGE gel (PG112, EpiZyme). The separated protein on the gel was transferred onto a polyvinylidene difluoride membrane (IPFL00010, Millipore), followed by blocking using 5% bovine serum albumin (BSA, 11413164, Fisher Scientific) in Tris borate EDTA (TBE) and staining with primary antibodies at 4 °C overnight. The membranes were subsequently stained with specific species-matched secondary antibodies conjugated with IRDye dissolved in 3% BSA in TBE for 1 h at room temperature. For visualization of positive signals, the Odyssey CLx system (LI-COR) was used. The positive signals were quantified using an Image Studio v.3.1 (LI-COR). β-Actin was used as the loading control for all blots. Primary and secondary antibodies were as follows: PI3K p85 (1:1,000, 4257, Cell Signaling Technology), phosphorylated PI3K p85 (1:1,000, 4228, Cell Signaling Technology), AKT (1:1,000, 9272, Cell Signaling Technology), phosphorylated AKT (1:1,000, 4051, Cell Signaling Technology), mTOR (1:1,000, 2972, Cell Signaling Technology), phosphorylated mTOR (1:1,000, 2971, Cell Signaling Technology), β-actin (1:1,000, 3700, Cell Signaling Technology), rabbit anti-mouse beta-actin polyclonal (1:1,000, 20536-1-AP, Proteintech), donkey anti-rabbit IRDye 680RD (1:15,000, 926–68073, LI-COR Biosciences) and donkey anti-mouse IRDye 800CW (1:15,000, 926–32212, LI-COR Biosciences) antibodies. For some of the samples, primary antibodies including rabbit anti-PI3K p85 alpha (1:1,000, ab191606, Abcam), rabbit anti-phosphorylated PI3K p85 alpha (1:1,000, ab182651, Abcam), rabbit anti-AKT (1:1,000, ab8805, Abcam), rabbit anti-phosphorylated AKT (1:1,000, ab38449, Abcam), rabbit anti-GLUT1 (1:1,000, ab115730, Abcam) and mouse anti-GAPDH (1:1,000, A01020, Abbkine) antibodies were used. Secondary antibodies including goat anti-mouse HRP-conjugated IgG (1:5,000, AS003, ABclonal) and goat anti-rabbit HRP-conjugated IgG (1:5,000, AS014, ABclonal) antibodies were used. Target proteins were visualized using the EZ ECL pico luminescence reagent (AP34L025, Life-iLab) with the Molecular Imager ChemiDoc XRS System (Bio-Rad). Full blot gels are provided in Supplementary Fig. 2.

### Human study

All of the human studies were approved by the Ethical Review Committee in the Qilu Hospital, Shandong University, Shandong Province, China (KYLL-202011-011-02), and informed consent for human research and the publication of the PET–CT images was obtained from all of the participants. Healthy volunteers including 3 men (body mass index, 23.0 ± 0.4 kg m^−2^) and 3 women (body mass index, 23.1 ± 0.5 kg m^−2^) aged between 22 and 25 years were recruited for the cold-exposure studies. Before cold exposure, all of the volunteers were fasted overnight and kept at 28 °C for 1 h. After 1 h, ^18^F-FDG at a dose of 0.1 mCi kg^−1^ was intravenously injected into each of the volunteers, followed by a PET scan (GE Discovery STE16, GE Healthcare). The healthy male and female individuals with T-shirts and shorts were exposed to a mildly cold ambient temperature of 16 °C for 2–6 h per day for consecutive 14 days. On day 13, all of the volunteers were fasted overnight and continued cold exposure for 2 h the next morning. PET scans were performed while the individuals intermittently soaked their feet in ice water. PET scan images were collected.

For patients with cancer, an 18-year-old female patient with Hodgkin’s lymphoma participated in a pilot study during the intervals of the fifth cycle of combination chemotherapy of adriamycin, bleomycin, vinblastine and dacarbazine for 5 cycles. The cold ambient temperature was approximately 22 °C regulated by air-conditioning for 1 week and the patient wore light clothing. The patient fasted overnight before PET–CT examination. The warm temperature exposure was about 28–30 °C, regulated by air-conditioning for 4 days. On day 4 after warm exposure, the patient was examined using PET–CT after overnight fasting. An experienced nuclear-medicine physician examined and analysed the PET and PET–CT images, and BAT activation and tumour ^18^F-FDG absorption were quantified.

### Statistical analysis

Sample numbers, the number of biological replicates and statistical analysis methods are provided in the figure legends. Statistical analysis was performed using Microsoft 365 Excel, GraphPad Prism v.9.2.0, and R (v.4.0.3). Data are presented as the means of determinants ± s.e.m. For RNA-seq, *P* values were calculated using the Wald test. *P* < 0.05 was deemed to be statistically significant.

### Reporting summary

Further information on research design is available in the Nature Research Reporting Summary linked to this article.

## Online content

Any methods, additional references, Nature Research reporting summaries, source data, extended data, supplementary information, acknowledgements, peer review information; details of author contributions and competing interests; and statements of data and code availability are available at 10.1038/s41586-022-05030-3.

## Supplementary information

Supplementary Figs. 1 and 2 Supplementary Fig. 1: FACS gating strategy for Extended Data Fig. 1c, d. Supplementary Fig. 2: full scan gels for western blotting analysis for Fig. 4e, Fig. 5c and Extended Data Fig. 5l.

Reporting Summary

Supplementary Table 1 A list of nucleotide sequences of primers used for qPCR in this study.

## Data Availability

All data of RNA-seq and metabolomics have been deposited at the NCBI Gene Expression Omnibus (GSE203148) and EMBL-EBI’s MetaboLights (MTBLS4856). Full scans for all western blots are provided in the Supplementary Information.  Source data are provided with this paper.

## Source data

Source Data Fig. 1
Source Data Fig. 2
Source Data Fig. 3
Source Data Fig. 4
Source Data Fig. 5
Source Data Fig. 6
Source Data Extended Data Fig. 1
Source Data Extended Data Fig. 2
Source Data Extended Data Fig. 3
Source Data Extended Data Fig. 4
Source Data Extended Data Fig. 5
Source Data Extended Data Fig. 6
Source Data Extended Data Fig. 7
