# Supplementary material for: Brown-fat-mediated tumour suppression by cold-altered global metabolism
Source: Nature. 2022 Aug 3;608(7922):421–8. doi: 10.1038/s41586-022-05030-3 (PMC9365697; doi:10.1038/s41586-022-05030-3)

nature portfolio

https://doi.org/10.1038/s41586-022-05030-3

Supplementary information

Brown-fat-mediated tumour suppression by cold-altered global metabolism

In the format provided by the authors and unedited

Supplementary Figure 1-Extended Data Fig. 1c, d:
Gating strategies used for Extended Data Fig. 1c, d.
FSC/SSC gate for the elimination of debris followed by a gate for GFP+ tumor cells isolation.
Further, PI-Width/PI-Area was used for doublet exclusion for PI histograms.

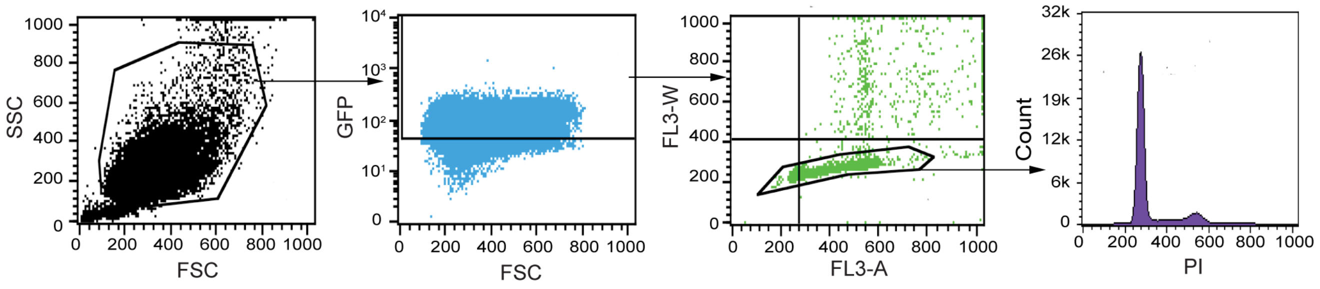

Supplementary Figure 2-Fig.4e:
Raw data of gels for immunoblots.
Whole gel images. Full scans of immunoblots in Fig.4e.
Boxes imply area shown in the Figure.

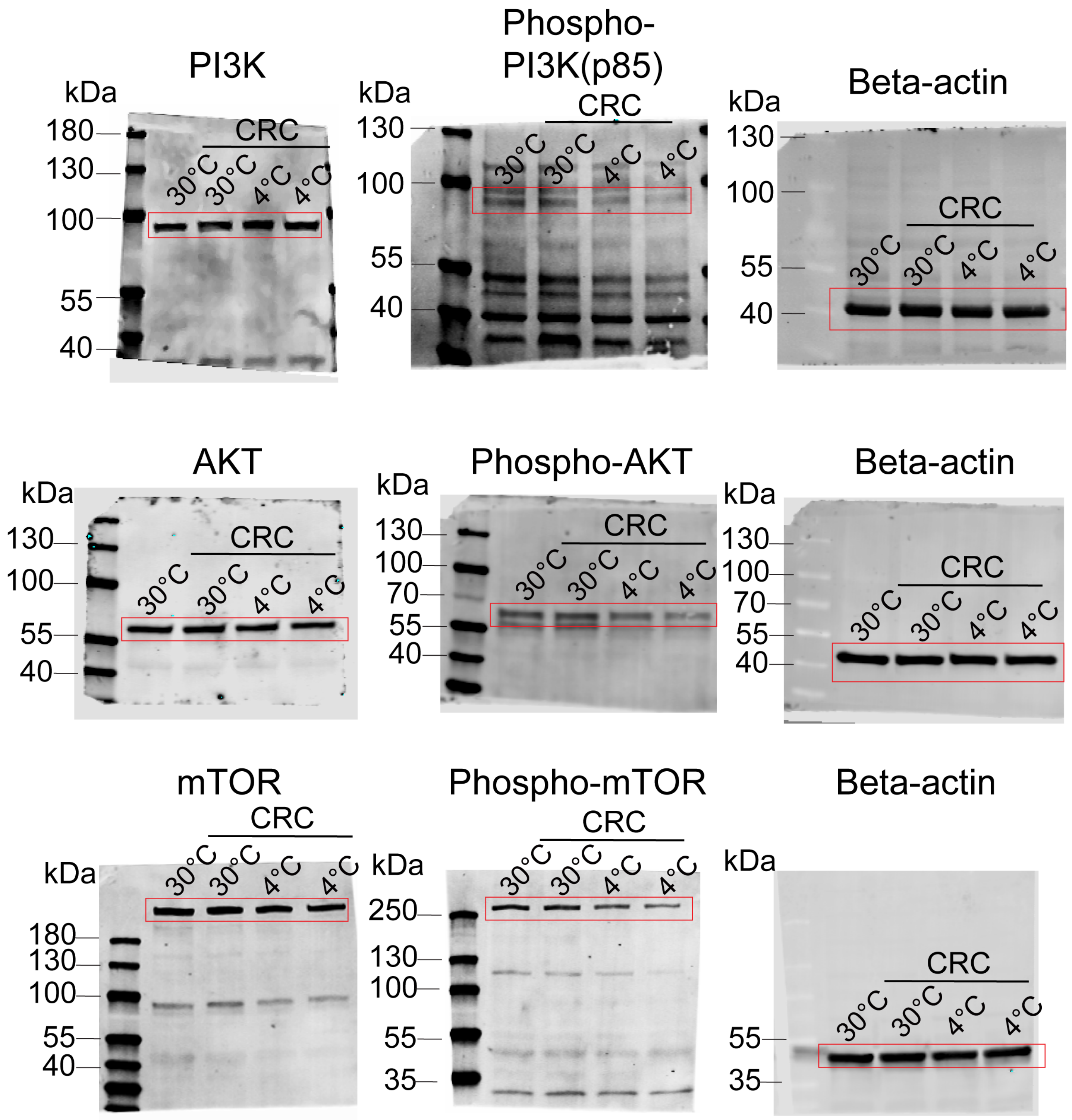

Supplementary Figure 2-Fig.5c:
Raw data of gels for immunoblots.
Whole gel images. Full scans of immunoblots in Fig.5c
Boxes imply area shown in the Figure.

PI3K

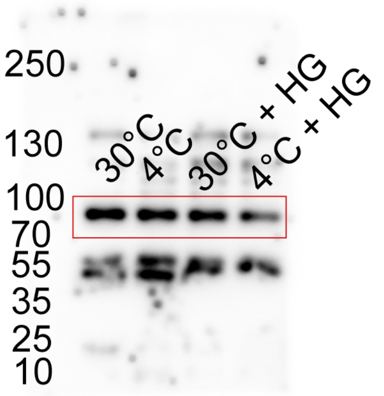

Phospho-PI3K(p85)

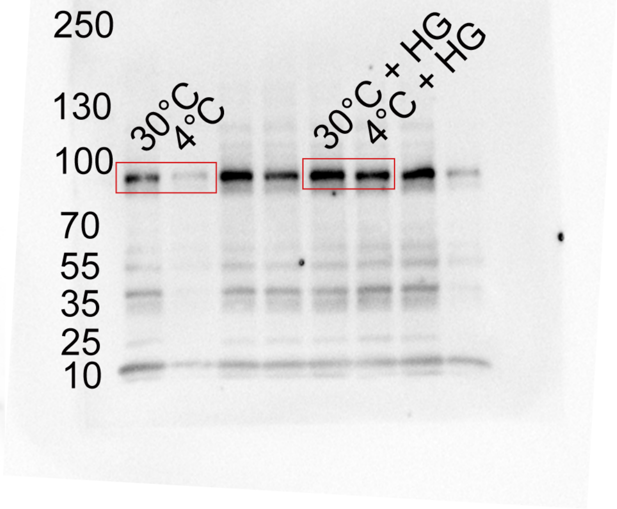

AKT

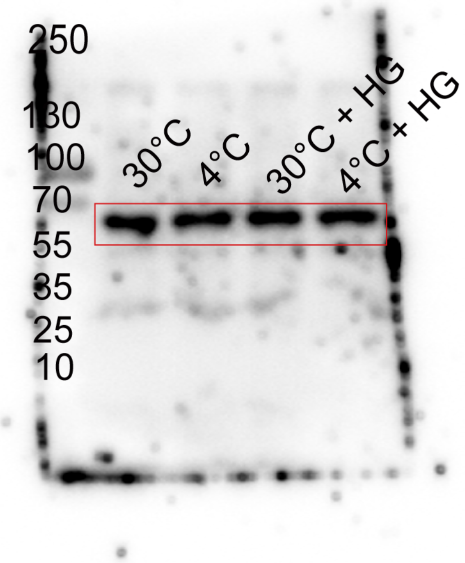

Phospho-AKT

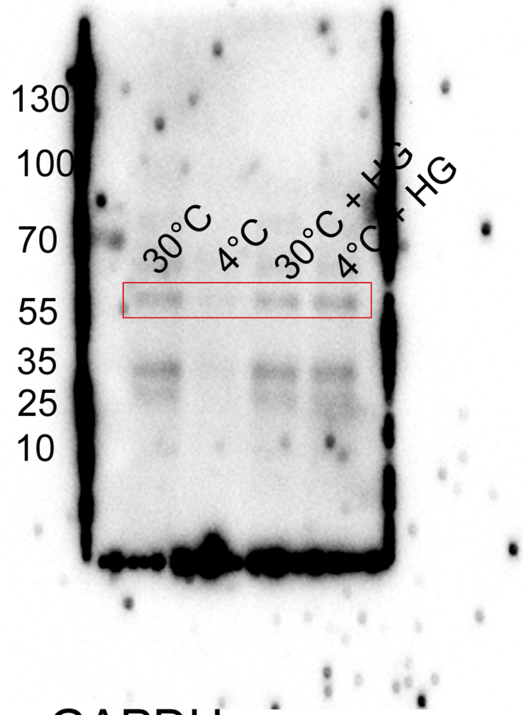

GLUT1

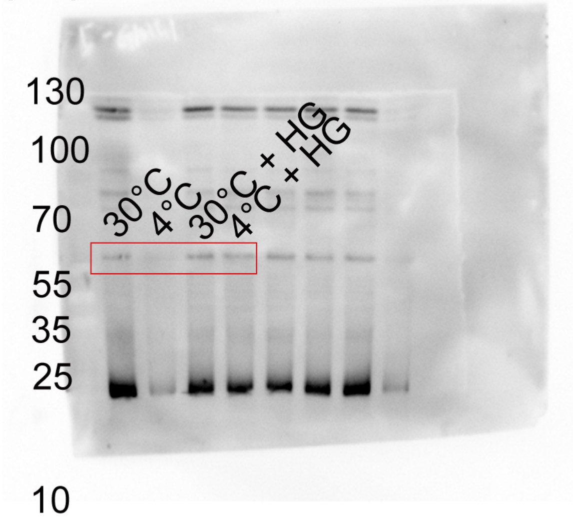

GAPDH

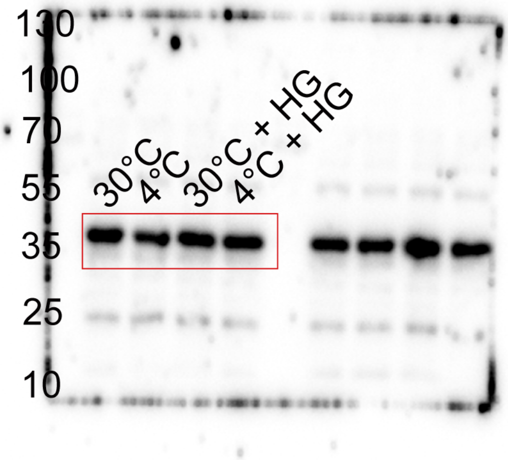

Supplementary Figure 2-Extended Data Fig 5l:
Raw data of gels for immunoblots.
Whole gel images. Full scans of immunoblots in Extended Data Fig 5l.
Boxes imply area shown in the Figure.

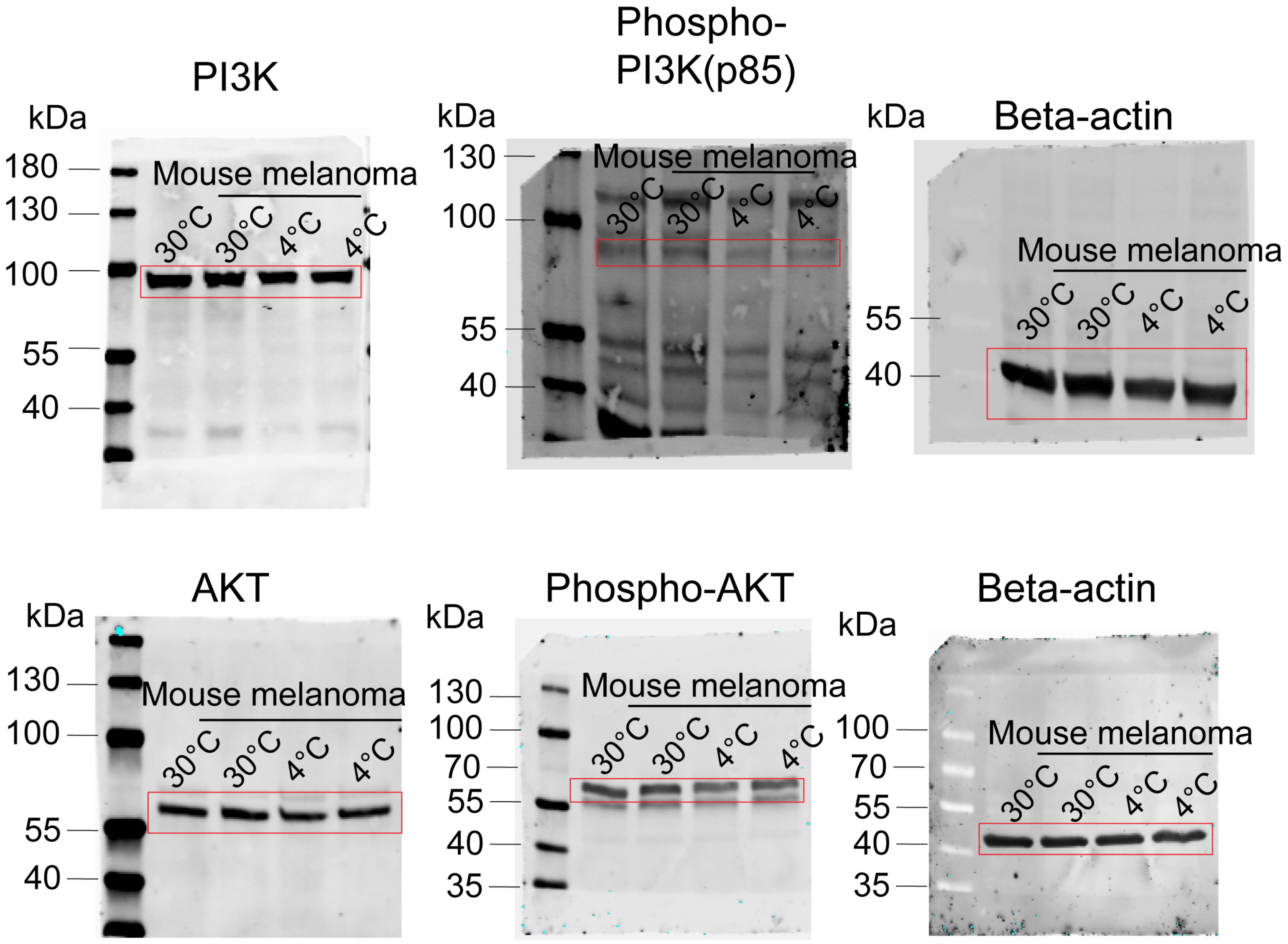

Supplement: Supplementary file 1 — Supplementary Fig. 1: FACS gating strategy for Extended Data Fig. 1c, d. Supplementary Fig. 2: full scan gels for western blotting analysis for Fig. 4e, Fig. 5c and Extended Data Fig. 5l. [file 41586_2022_5030_MOESM1_ESM.pdf]
