# Supplementary material for: Brown-fat-mediated tumour suppression by cold-altered global metabolism
Source: Nature. 2022 Aug 3;608(7922):421–8. doi: 10.1038/s41586-022-05030-3 (PMC9365697; doi:10.1038/s41586-022-05030-3)
Supplement: Supplementary file 2 — Reporting Summary [file 41586_2022_5030_MOESM2_ESM.pdf]

nature research

Corresponding author(s): Yihai Cao

Last updated by author(s): May 24, 2022

Reporting Summary

Nature Research wishes to improve the reproducibility of the work that we publish. This form provides structure for consistency and transparency in reporting. For further information on Nature Research policies, see our Editorial Policies and the Editorial Policy Checklist.

Statistics

For all statistical analyses, confirm that the following items are present in the figure legend, table legend, main text, or Methods section.

n/a	Confirmed	
☐	☒	The exact sample size (*n*) for each experimental group/condition, given as a discrete number and unit of measurement
☐	☒	A statement on whether measurements were taken from distinct samples or whether the same sample was measured repeatedly
☐	☒	The statistical test(s) used AND whether they are one- or two-sided<br>*Only common tests should be described solely by name; describe more complex techniques in the Methods section.*
☐	☒	A description of all covariates tested
☐	☒	A description of any assumptions or corrections, such as tests of normality and adjustment for multiple comparisons
☐	☒	A full description of the statistical parameters including central tendency (e.g. means) or other basic estimates (e.g. regression coefficient) AND variation (e.g. standard deviation) or associated estimates of uncertainty (e.g. confidence intervals)
☐	☒	For null hypothesis testing, the test statistic (e.g. *F*, *t*, *r*) with confidence intervals, effect sizes, degrees of freedom and *P* value noted<br>*Give P values as exact values whenever suitable.*
☒	☐	For Bayesian analysis, information on the choice of priors and Markov chain Monte Carlo settings
☒	☐	For hierarchical and complex designs, identification of the appropriate level for tests and full reporting of outcomes
☒	☐	Estimates of effect sizes (e.g. Cohen's *d*, Pearson's *r*), indicating how they were calculated

Our web collection on statistics for biologists contains articles on many of the points above.

Software and code

Policy information about availability of computer code

Data collection	-Immunofluorescence images were captured using a camera (Nikon, DS-QilMC) with NIS-Elements D3.2 (Nikon) software and quantified using Adobe Photoshop software CS5 extended (Adobe) and ImageJ Fiji.<br>- The confocal images were recorded using the camera 1 of a Nikon C1 confocal microscope with EZ-C1 3.91 software (Nikon) and quantified using Adobe Photoshop software CS5 extended (Adobe) and ImageJ Fiji.<br>- For H&E staining, the image was captured by a light microscope (Nikon Eclipse TS100) with the camera (DS-Fil, Nikon) using NIS-Elements F 3.0 software (Nikon), and quantified using Adobe Photoshop software CS5 extended (Adobe) and ImageJ Fiji.<br>-Flow cytometry files were collected using BD FACSCalibur™ Flow Cytometer (BD Bioscience) using a CellQuest Pro software (version 6.0, BD Bioscience).<br>-Immunoblots files were collected using an Odyssey CLx system (LI-COR) with an Image Studio version 3.1 (LI-COR) or using a Molecular Imager ChemiDoc XRS System (Bio-Rad).<br>-RNA-sequencing results were collected using Illumina Miseq sequencer.<br>-The mass spectrometry metabolomics data were collected using Agilent MassHunter Qualitative Analysis software version b.06.00 (Agilent Technologies).<br>-Thermal images captured by infrared thermal imager were quantified using Fotric AnalyzIR software (no version number).<br>-PET-CT images were quantified using Inveon MicroPET/CT system includes Inveon Acquisition workplace and Inveon Research workplace software Version 4.2 (Siemens Medical Solution, California, USA).
Data analysis	Microsoft 365 Excel; GraphPad Prism 9.2.0; FeatureCounts (v2.0.0); R (v4.0.3); DESeq2 (v1.30.0); GSEA (v4.1.0); BD Cell Quest Pro version 6.0

For manuscripts utilizing custom algorithms or software that are central to the research but not yet described in published literature, software must be made available to editors and reviewers. We strongly encourage code deposition in a community repository (e.g. GitHub). See the Nature Research guidelines for submitting code & software for further information.

Data

Policy information about availability of data

All manuscripts must include a data availability statement. This statement should provide the following information, where applicable:

- Accession codes, unique identifiers, or web links for publicly available datasets
- A list of figures that have associated raw data
- A description of any restrictions on data availability

All RNA-sequencing and metabolomics data generated in this study have been deposited in NCBI's Gene Expression Omnibus (GSE203148) and EMBL-EBI's MetaboLights (MTBLS4856). Full scans for all western blots are provided in the Supplementary Information. Source data are provided in this paper

Field-specific reporting

Please select the one below that is the best fit for your research. If you are not sure, read the appropriate sections before making your selection.

☒ Life sciences ☐ Behavioural & social sciences ☐ Ecological, evolutionary & environmental sciences

For a reference copy of the document with all sections, see nature.com/documents/nr-reporting-summary-flat.pdf

Life sciences study design

All studies must disclose on these points even when the disclosure is negative.

Sample size

No statistical calculations were used to predetermine sample size for in vivo experiments. Sample sizes are indicated in Figure legends. Our previous tumor studies [1-3] determined the sample sizes by the extent and consistency of measurable differences to ensure statistical and biological significance. Besides, an optimal number of animals were employed for the aspect of the 3R principle for animal ethical permission [4].

[1] Xiaoting Sun et al. Inflammatory cell-derived CXCL3 promotes pancreatic cancer metastasis through a novel myofibroblast-hijacked cancer escape mechanism. Gut. 2022 Jan;71(1):129-147.
[2] Kayoko Hosaka et al, Therapeutic paradigm of dual targeting VEGF and PDGF for effectively treating FGF-2 off-target tumors. Nat Commun. 2020 Jul 24;11(1):3704.
[3] Hideki Iwamoto et al. Cancer lipid metabolism confers antiangiogenic drug resistance. Cell Metab. 2018 Jul 3;28(1):104-117.e5.
[4] Swedish 3R-center, https://jordbruksverket.se/languages/english/the-swedish-3rs-center

Data exclusions

No data were excluded.

Replication

Murine studies: Experiments were performed at least twice. The fundamental tumor experiments for different temperature exposures were performed at least twice in multi-institution and were reproducible. Omics studies: Quality samples control was performed before subjecting those analyses. RNA sequencing for Fig.4 was performed once using two to three independent biological replicants because of further validation using unbiased alternative and quantitative assessment, such as qPCR. Analysis for metabolites for Fig. 4 was performed once using four independent biological replicants and was validated by another cohort for ED Fig 6. Clinical studies: individuals were considered replicated. The PET-CT scan was performed twice and three times for healthy individuals and the patient.

Randomization

Murine studies: Age, gender, and background-matched mice were randomly allocated into the groups for all animal experiments. Clinical study: Age, gender, and BMI-matched healthy individuals groups underwent the same trials. For a cancer patient, randomization was not relevant.

Blinding

Murine studies: Blinding was not performed for experiments involving different temperature exposure. Because the animals were kept in special temperature-controlled rooms or chambers during tumor study, it was not possible to hide the animal identity. PET-CT technologist at Fudan University in China was blinded to the group allocation.
Omics studies: The authors who were blinded to experimental groups performed the metabolite analyses in mouse tissues. Staff at the Fudan University in China who were blinded to the experimental groups did RNA sequencing and library constructions.
Several co-authors performed a non-blinded study at the Department of Nuclear Medicine, Qilu Hospital, China, for PET-CT scans and the analysis of healthy individuals and the cancer patient. An experienced nuclear medicine physician analyzed the PET or PET-CT images and BAT activation and tumor 18F-FDG absorption. It was impossible for them to blindly carry out the study because they were involved the recruitment and treatment of the healthy volunteers and the patient.

Reporting for specific materials, systems and methods

We require information from authors about some types of materials, experimental systems and methods used in many studies. Here, indicate whether each material, system or method listed is relevant to your study. If you are not sure if a list item applies to your research, read the appropriate section before selecting a response.

Materials & experimental systems

n/a	Involved in the study
☐	☒ Antibodies
☐	☒ Eukaryotic cell lines
☒	☐ Palaeontology and archaeology
☐	☒ Animals and other organisms
☐	☒ Human research participants
☒	☐ Clinical data
☒	☐ Dual use research of concern

Methods

n/a	Involved in the study
☒	☐ ChIP-seq
☐	☒ Flow cytometry
☒	☐ MRI-based neuroimaging

Antibodies

Antibodies used

Information for antibodies for this study are,
- Goat IgG anti-mouse CD31 polyclonal antibody affinity-purified from goat sera (1:200; AF3628; R&D)
- Rabbit anti-mouse Cleaved Caspase 3 polyclonal antibody (1:200; 9661; Cell Signaling)
- Rabbit anti-mouse Ki67 polyclonal antibody (1:100; NB110-89719; Novus biologicals)
- Rat anti-mouse Ki67 antibody (SolA15; 1:100; 14-5698-82; Thermo Fisher Scientific)
- Rabbit anti-mouse FSP1/S100A4 polyclonal antibody (1:300; 07-2274; Merck)
- Rabbit anti-mouse Iba1 polyclonal antibody (1:200; 019-19741; FUJIFILM Wako)
- Rabbit anti-mouse CD45 polyclonal antibody (1:200; ab10558; abcam)
- Rabbit anti-mouse polyclonal CA9 (1:100; NB100-417; Novus biologicals)
- Rabbit anti-mouse GLUT1 antibody (EPR3915; 1:1000; ab115730; abcam)
- Rabbit anti-mouse UCP1 polyclonal antibody (1:200; ab 10983; abcam)
- Rabbit anti-mouse COX4 polyclonal antibody (1:300; GTX114330, GeneTex)
- Guinea pig anti-mouse Perilipin polyclonal antibody (1:300; 20R-PP004, Fitzgerald Industries)
- Rabbit anti-mouse PI3K p85 antibody (19H8; 1:1000; 4257; Cell Signaling Technology)
- Rabbit anti-mouse PI3K p85 alpha antibody (EPR18702; 1:1000; ab191606, abcam)
- Rabbit anti-mouse phospho-PI3K p85 polyclonal antibody (1:1000; 4228; Cell Signaling Technology)
- Rabbit anti-mouse phospho-PI3K p85 alpha polyclonal antibody (1:1000; ab182651, abcam)
- Rabbit anti-mouse AKT polyclonal antibody (1:1000; 9272; Cell Signaling Technology)
- Rabbit anti-mouse AKT polyclonal antibody (1:1000; ab8805, abcam)
- Mouse anti-mouse phospho-AKT antibody (587F11; 1:1000; 4051; Cell Signaling Technology)
- Rabbit anti-mouse phospho-AKT polyclonal antibody (1:1000; ab38449; abcam)
- Rabbit anti-mouse mTOR polyclonal antibody (1:1000; 2972; Cell Signaling Technology)
- Rabbit anti-mouse phospho-mTOR polyclonal antibody (1:1000; 2971; Cell Signaling Technology)
- Mouse anti-mouse beta-actin antibody (8H10D10; 1:1000; 3700; Cell Signaling Technology)
- Rabbit anti-mouse beta-actin polyclonal antibody (1:1000; 20536-1-AP; Proteintech)
- Mouse anti-mouse GAPDH antibody (A01020; 1:1000; A01020, now ABL1020; Abbkine)
- Alexa Fluor 555-labeled donkey anti-goat antibody (1:300; A21432; Thermo Fisher Scientific)
- Alexa Fluor 488-labeled donkey anti-rabbit antibody (1:300; A21206, Thermo Fisher Scientific)
- Alexa Fluor 555 goat anti-rabbit antibody (1:300; A21428, Thermo Fisher Scientific)
- Alexa Fluor 647 goat anti-guinea pig antibody (1:200; A-21450, Thermo Fisher Scientific)
- HRP-conjugated goat anti-mouse IgG antibody (1:5000; AS003; ABclonal)
- HRP-conjugated goat anti-rabbit IgG antibody (1:5000; AS014; ABclonal)
- Donkey anti-Rabbit IRDye 680RD antibody (1:15000, 926-68073, LI-COR Biosciences)
- Donkey anti-mouse IRDye 800CW antibody (1:15000, 926-32212, LI-COR Biosciences)

Validation

All antibodies used in this study were validated for the application and species by their manufacturers. The link is listed below.

- Goat IgG anti-mouse CD31 polyclonal antibody affinity-purified from goat sera (1:200; AF3628; R&D)
https://www.rndsystems.com/products/mouse-rat-cd31-pecam-1-antibody_af3628
- Rabbit anti-mouse Ki67 polyclonal antibody (1:100; NB110-89719; Novus biologicals)
https://www.novusbio.com/products/ki67-mki67-antibody_nb110-89719
- Rat anti-mouse Ki67 antibody (SolA15; 1:100; 14-5698-82; Thermo Fisher Scientific)
https://www.thermofisher.com/antibody/product/Ki-67-Antibody-clone-SolA15-Monoclonal/14-5698-82
- Rabbit anti-mouse FSP1/S100A4 polyclonal antibody (1:300; 07-2274; Merck)
https://www.merckmillipore.com/SE/en/product/Anti-FSP1-S100A4-Antibody,MM_NF-07-2274
- Rabbit anti-mouse Iba1 polyclonal antibody (1:200; 019-19741; FUJIFILM Wako)
https://labchem-wako.fujifilm.com/us/product/detail/W01W0101-1974.html
- Rabbit anti-mouse CD45 polyclonal antibody (1:200; ab10558; abcam)
https://www.abcam.com/cd45-antibody-ab10558.html
- Rabbit anti-mouse polyclonal CA9 (1:100; NB100-417; Novus biologicals)
https://www.novusbio.com/products/carbonic-anhydrase-ix-ca9-antibody_nb100-417
- Rabbit anti-mouse GLUT1 antibody (EPR3915; 1:1000; ab115730; abcam)
https://www.abcam.com/akt-phospho-t308-antibody-ab38449.html
- Rabbit anti-mouse UCP1 polyclonal antibody (1:200; ab 10983; abcam)
https://www.abcam.com/ucp1-antibody-ab10983.html
- Rabbit anti-mouse COX4 polyclonal antibody (1:300; GTX114330, GeneTex)
https://www.genetex.com/Product/Detail/COX4-antibody/GTX114330
- Guinea pig anti-mouse Perilipin polyclonal antibody (1:300; 20R-PP004, Fitzgerald Industries)

https://www.fitzgerald-fii.com/perilipin-antibody-20r-pp004.html
- Rabbit anti-mouse PI3K p85 antibody (19H8; 1:1000; 4257; Cell Signaling Technology)
https://www.cellsignal.com/products/primary-antibodies/pi3-kinase-p85-19h8-rabbit-mab/4257
- Rabbit anti-mouse PI3K p85 alpha antibody (EPR18702; 1:1000; ab191606, abcam)
https://www.abcam.com/pi-3-kinase-p85-alpha-antibody-epr18702-ab191606.html

- Rabbit anti-mouse phospho-PI3K p85 polyclonal antibody (1:1000; 4228; Cell Signaling Technology)
https://www.cellsignal.com/products/primary-antibodies/phospho-pi3-kinase-p85-tyr458-p55-tyr199-antibody/4228
- Rabbit anti-mouse phospho-PI3K p85 alpha polyclonal antibody (1:1000; ab182651, abcam)
https://www.abcam.com/pi-3-kinase-p85-alpha-phospho-y607-antibody-ab182651.html
- Rabbit anti-mouse AKT polyclonal antibody (1:1000; 9272; Cell Signaling Technology)
https://www.cellsignal.com/products/primary-antibodies/akt-antibody/9272
- Rabbit anti-mouse AKT polyclonal antibody (1:1000; ab8805, abcam)
https://www.abcam.com/pan-akt-antibody-ab8805.html
- Mouse anti-mouse phospho-AKT antibody (587F11; 1:1000; 4051; Cell Signaling Technology)
https://www.cellsignal.com/products/primary-antibodies/phospho-akt-ser473-587f11-mouse-mab/4051
- Rabbit anti-mouse phospho-AKT polyclonal antibody (1:1000; ab38449; abcam)
https://www.abcam.com/akt-phospho-t308-antibody-ab38449.html
- Rabbit anti-mouse mTOR polyclonal antibody (1:1000; 2972; Cell Signaling Technology)
https://www.cellsignal.com/products/primary-antibodies/mtor-antibody/2972
- Rabbit anti-mouse phospho-mTOR polyclonal antibody (1:1000; 2971; Cell Signaling Technology)
https://www.cellsignal.com/products/primary-antibodies/phospho-mtor-ser2448-antibody/2971
- Mouse anti-mouse beta-actin antibody (8H10D10; 1:1000; 3700; Cell Signaling Technology)
https://www.cellsignal.com/products/primary-antibodies/b-actin-8h10d10-mouse-mab/3700
- Rabbit anti-mouse beta-actin polyclonal antibody (1:1000; 20536-1-AP; Proteintech)
https://www.ptglab.com/products/ACTB-Antibody-20536-1-AP.htm
- Mouse anti-mouse GAPDH antibody (A01020; 1:1000; ABL1020; Abbkine)
https://www.abbkine.cn/product/abl1020/
- Alexa Fluor 555 labeled donkey anti-goat antibody (1:300; A21432; Thermo Fisher Scientific)
https://www.thermofisher.com/antibody/product/Donkey-anti-Goat-IgG-H-L-Cross-Adsorbed-Secondary-Antibody-Polyclonal/A-21432
- Alexa Fluor 488-labeled donkey anti-rabbit antibody (1:300; A21206, Thermo Fisher Scientific)
https://www.thermofisher.com/antibody/product/Donkey-anti-Rabbit-IgG-H-L-Highly-Cross-Adsorbed-Secondary-Antibody-Polyclonal/A-21206
- Alexa Fluor 555-labeled goat anti-rat antibody (1:300; A21434; Thermo Fisher Scientific)
https://www.thermofisher.com/antibody/product/Goat-anti-Rat-IgG-H-L-Cross-Adsorbed-Secondary-Antibody-Polyclonal/A-21434
-Alexa Fluor 555 goat anti-rabbit antibody (1:300; A21428, Thermo Fisher Scientific)
https://www.thermofisher.com/antibody/product/Goat-anti-Rabbit-IgG-H-L-Cross-Adsorbed-Secondary-Antibody-Polyclonal/A-21428
- Alexa Fluor 647 goat anti-guinea pig antibody (1:200; A-21450, Thermo Fisher Scientific)
https://www.thermofisher.com/antibody/product/Goat-anti-Guinea-Pig-IgG-H-L-Highly-Cross-Adsorbed-Secondary-Antibody-Polyclonal/A-21450
- HRP-conjugated goat anti-mouse IgG antibody (1:5000; AS003; ABclonal)
https://abclonal.com/catalog-antibodies/HRPGoatAntiMouseIgGHL/AS003
- HRP-conjugated goat anti-rabbit IgG antibody (1:5000; AS014; ABclonal)
https://abclonal.com/catalog-antibodies/HRPGoatAntiRabbitIgGHL/AS014
- Donkey anti-Rabbit IRDye 680RD antibody (1:15000, 926-68073, LI-COR Biosciences)
https://www.licor.com/documents/98163zqw7d2pmt0fkihkn11gtozr41vn
- Donkey anti-mouse IRDye 800CW antibody (1:15000, 926-32212, LI-COR Biosciences)
https://www.licor.com/documents/ekvg3zmbe83nlayg2pq84lz6jdu1i854

Eukaryotic cell lines

Policy information about cell lines

Cell line source(s)	-Murine MC-38 colon adenocarcinoma cell line is available from Kerafast (Boston, MA, USA).<br>-Murine pancreatic cancer cell line Panc02 is available from Creative Biolabs (Shirley, NY, USA).<br>-Murine E0771 breast cancer cell line was purchased from CH3 BioSystems (Amherst, NY, USA).<br>-Human colorectal carcinoma HCT 116 tumor cells, human pancreas duct epithelioid carcinoma PANC-1, murine fibrosarcoma T241, and murine melanoma B16-F10 were purchased from ATCC.
Authentication	All cell lines were not authenticated.
Mycoplasma contamination	All cell lines used in our study were negative for mycoplasma as detected by a mycroplasma kit (Cat. No. LT07-318, Lonza).
Commonly misidentified lines<br>(See ICLAC register)	No commonly misidentified cell line was used.

Animals and other organisms

Policy information about studies involving animals; ARRIVE guidelines recommended for reporting animal research

Laboratory animals	This study used laboratory mice for in vivo animal model.<br>Wild type C57Bl/6 and immunodeficient SCID mice were obtained from the breeding unit at the Department of Microbiology, Tumor

	and Cell Biology, Karolinska Institute, Stockholm, Sweden and from the Model Animal Research Center of Nanjing University. FVB/NJGpt-Tg(MMTV-PyMT)/Gpt mice were purchased from the GemPharmatech, Nanjing, China.<br>C57Bl/6J background-ApcMin/+ mice were obtained from the Jackson Laboratory. Ucp1-/- mice in the C57BL/6 background were purchased from the Jackson Laboratory.
Wild animals	This project did not use wild animals.
Field-collected samples	This study did not collect samples from the field.
Ethics oversight	All mouse studies were approved by the North Stockholm Animal Ethical Committee, Stockholm, Sweden, or by the Animal Experimental Ethical Committee of the Fudan University, Shanghai, China.

Note that full information on the approval of the study protocol must also be provided in the manuscript.

Human research participants

Policy information about studies involving human research participants

Population characteristics	A total of six healthy volunteers, three males (BMI 23.0 ± 0.4 kg/m2) and three females (BMI 23.1 ± 0.5 kg/m2), aged between 22 and 25 years old, took part in this study. An eighteen-year-old female cancer patient with Hodgkin's lymphoma who is under chemotherapy participated in this study. The detail of the chemotherapy described in Methods.
Recruitment	For healthy individuals, the volunteers were recruited using the call for trial participation by the group chat of Fudan University. For cancer patients, the doctor and the medical coordinator in the Department of Nuclear Medicine, Qilu Hospital, China, explained to the cancer patient and her family the detailed information on this study, the cold exposure procedure, the potential risks and benefits of the cold exposure, and the rights to withdraw from the empirical study at any time. All participants had written informed consent and were consulted for this study. The potential self-selection bias for the selection was not observed.
Ethics oversight	Informed consent was obtained from all participants, including the patient. All human studies were approved by the Ethical Review Committee in the Qilu Hospital, Shandong University, Shandong Province, China.

Note that full information on the approval of the study protocol must also be provided in the manuscript.

Flow Cytometry

Plots

Confirm that:

☒ The axis labels state the marker and fluorochrome used (e.g. CD4-FITC).

☒ The axis scales are clearly visible. Include numbers along axes only for bottom left plot of group (a 'group' is an analysis of identical markers).

☒ All plots are contour plots with outliers or pseudocolor plots.

☒ A numerical value for number of cells or percentage (with statistics) is provided.

Methodology

Sample preparation	Manually dissected tumors were cut and enzymatically digested using 0.15% collagenase I (17100-017, Gibco) and collagenase II (17101015, Gibco) in PBS for 40 min at 37 °C, and centrifugated at 1500 rpm, 4 °C for 10 min. The pellets were resuspended with PBS containing 1% FBS. The single-cell was obtained by filtration using 70-µm- cell strainers followed by centrifugation at 1300 rpm, 4 °C for 10 min. The pellets were fixed using 1% PFA in PBS for 15 min at room temperature and washed in PBS. After centrifugation at 1300 rpm, 4 °C for 10 min, the cell pellets were further resuspended in PBS, and cold 70% ethanol was subsequently added dropwise to the cell suspension while vertexing. The samples were stored at 4 °C for further analysis. For the DNA content, fixed single-cell suspensions in PBS were incubated with 20 mg/ml propidium iodide (PI) (P3566, Invitrogen) and 100 mg/ml RNase (EN0531, Thermo Scientific) final on ice for 30 min, followed by analysis on the flow cytometer. We describe the detail in the Methods section.
Instrument	BD FACS Calibur
Software	CellQuest Pro ver 6.0 software
Cell population abundance	We targeted PI and GFP positive cells and GFP positive cells are the main populations in the samples.
Gating strategy	FSC/SSC gate for the elimination of debris followed by a gate for GFP+ tumor cells isolation.<br>Further, PI-Width/PI-Area was used for doublet exclusion for PI histograms.

☒ Tick this box to confirm that a figure exemplifying the gating strategy is provided in the Supplementary Information.
