# Supplementary material for: Brown-fat-mediated tumour suppression by cold-altered global metabolism
Source: Nature. 2022 Aug 3;608(7922):421–8. doi: 10.1038/s41586-022-05030-3 (PMC9365697; doi:10.1038/s41586-022-05030-3)
Supplement: Supplementary file 3 — A list of nucleotide sequences of primers used for qPCR in this study. [file 41586_2022_5030_MOESM3_ESM.docx]

| **Supplementary Table 1 Nucleotide sequences of primers used for qPCR** |
| --- |

| ***Gene*** | **Species** | **Forward primer sequence** | **Reverse primer sequence** |
| --- | --- | --- | --- |

| *Glut1* | mouse | 5’-CAATGGCGGCGGTCCTATAA-3’ | 5’-AACGGACGCGCTGTAACTAT-3’ |
| --- | --- | --- | --- |
| *Glut4* | mouse | 5’-ACACTGGTCCTAGCTGTATTCT-3’ | 5’-CCAGCCACGTTGCATTGTA-3’ |
| *Glut7* | mouse | 5’-CACGCACTTTGAGCGACAC-3’ | 5’-CCCACTTATTGACCATCAGGC-3’ |
| *Pgam2* | mouse | 5’-TGGAACCAAGAGAACCGTTTC-3’ | 5’-TGGCATCTTTGATAGCGGTGG-3’ |
| *Gck* | mouse | 5’-TGAGCCGGATGCAGAAGGA-3’ | 5’-GCAACATCTTTACACTGGCCT-3’ |
| *Bpgm* | mouse | 5’-GGACCAGAAACTTAACAACGACG-3’ | 5’-CAGGCTGTGTGAATGGACCT-3’ |
| *Actin* | mouse | 5’-AGGCCCAGAGCAAGAGAGG-3’ | 5’-TACATGGCTGGGGTGTTGAA-3’ |
| *Gapdh* | mouse | 5’-CCAGCAAGGACACTGAGCAA-3’ | 5’-GGGATGGAAATTGTGAGGGA-3’ |
|  |  |  |  |
